# Incidence of Cutaneous Immune-Related Adverse Events and Outcomes in Immune Checkpoint Inhibitor-Containing Regimens: A Systematic Review and Meta-Analysis

**DOI:** 10.3390/cancers16020340

**Published:** 2024-01-13

**Authors:** Nina B. Curkovic, Kun Bai, Fei Ye, Douglas B. Johnson

**Affiliations:** 1 School of Medicine, Vanderbilt University, Nashville, TN 37232, USA; 2 Vanderbilt Ingram Cancer Center, Department of Biostatistics, Vanderbilt University Medical Center, Nashville, TN 37232, USA; 3 Vanderbilt Ingram Cancer Center, Department of Medicine, Vanderbilt University Medical Center, Nashville, TN 37232, USA; douglas.b.johnson@vumc.org

**Keywords:** immune checkpoint inhibitors, cutaneous immune-related adverse events, autoimmune toxicities, anti-angiogenic, chemotherapy, melanoma, renal cell carcinoma, non-small cell lung cancer, urothelial carcinoma, meta-analysis

## Abstract

**Simple Summary:**

Immune checkpoint inhibitors are increasingly being used in the treatment of a variety of cancers, both alone and in combination with other cancer therapies. Side effects often include skin reactions, which may occur more frequently in therapeutic regimens consisting of multiple immune checkpoint inhibitors. We conducted a systematic review and meta-analysis of clinical trials to better understand the frequency of skin reactions secondary to immune checkpoint inhibitors, known as cutaneous immune-related adverse events, across several different immune checkpoint inhibitor regimens, doses, and cancers. Our analysis provides benchmark incidence rates for cutaneous immune-related adverse events including pruritis, rash, and vitiligo, and validates previously reported links between the development of cutaneous immune-related adverse events and outcomes of therapy.

**Abstract:**

Immune checkpoint inhibitors (ICIs) are used to treat many cancers, and cutaneous immune-related adverse events (cirAEs) are among the most frequently encountered toxic effects. Understanding the incidence and prognostic associations of cirAEs is of importance as their uses in different settings, combinations, and tumor types expand. To evaluate the incidence of cirAEs and their association with outcome measures across a variety of ICI regimens and cancers, we performed a systematic review and meta-analysis of published trials of anti–programmed death-1/ligand-1 (PD-1/PD-L1) and anti–cytotoxic T lymphocyte antigen-4 (CTLA-4) ICIs, both alone and in combination with chemotherapy, antiangiogenic agents, or other ICIs in patients with melanoma, renal cell carcinoma, non-small cell lung cancer, and urothelial carcinoma. Key findings of our study include variable cirAE incidence among tumors and ICI regimens, positive association with increased cirAE incidence and response rate, as well as significant association between increased vitiligo incidence and overall survival. Across 174 studies, rash, pruritis, and vitiligo were the most reported cirAEs, with incidences of 16.7%, 18.0%, and 6.6%, respectively. Higher incidence of cirAEs was associated with ICI combination regimens and with CTLA-4-containing regimens, particularly with higher doses of ipilimumab, as compared to PD-1/L1 monotherapies. Outcome measures including response rate and progression-free survival were positively correlated with incidence of cirAEs. The response rate and incidence of pruritis, vitiligo, and rash were associated with expected rises in incidence of 0.17% (*p* = 0.0238), 0.40% (*p* = 0.0010), and 0.18% (*p* = 0.0413), respectively. Overall survival was positively correlated with the incidence of pruritis, vitiligo, and rash; this association was significant for vitiligo (*p* = 0.0483). Our analysis provides benchmark incidence rates for cirAEs and links cirAEs with favorable treatment outcomes at a study level across diverse solid tumors and multiple ICI regimens.

## 1. Introduction

Immune checkpoint inhibitors (ICIs) have proven to be effective therapies for a variety of cancer types. By targeting immune checkpoints with anti-PD-1, anti-PD-L1, anti-CTLA4, and, more recently, anti-LAG-3 agents, ICIs disinhibit the immune system to unleash anti-tumor immune responses. However, clinically significant off-target effects due to the generation of autoreactive T cells, termed immune-related adverse events (irAEs), are common and can affect virtually any organ [1]. Cutaneous immune-related adverse events (cirAES) are among the most frequently encountered irAEs, occurring at rates of 30–40% with anti-PD-1 monotherapy, with potentially higher incidence and severity with combination regimens [1,2]. The clinical presentation of cirAEs is variable and may range from common eczematous and lichenoid eruptions to, less commonly, bullous pemphigoid and reports of associated cutaneous infections [3].

Several retrospective studies have suggested that cirAEs are associated with improved responses and survival [4,5,6,7]. These associations may be explained by shared antigens between the skin and tumors; a study on non-small cell lung cancer (NSCLC) patients treated with anti-PD-1 therapy identified T cell antigens that were shared between tumor tissue and skin, highlighting a mechanism by which the development of cirAEs could be associated with therapeutic benefit [8]. ICI-induced vitiligo, a cirAE more commonly seen in patients with melanoma, has been suggested to occur via ICI-induced loss of immune privilege to normal melanocytes following the release of shared melanocytic antigens within destroyed melanoma tumor cells [9].

Importantly, therapeutic benefits may also arise from other patient and tumor-specific factors. PDL-1 expression, metastatic site, tumor mutational burden, tumor-infiltrating lymphocytes, and the gut microbiome have been implicated in influencing the efficacy of ICIs [10,11].The development of cirAEs may be just one aspect of developing more individualized treatment prognostications as more information regarding patient-specific factors becomes available for use in clinical practice.

Prior meta-analyses have predominantly examined cirAEs in association with ICI monotherapy across cancer types or combined ICI regimens in a single cancer type [12,13,14]. As ICIs are increasingly being used in combination with multiple distinct immune and non-immune-based regimens, the incidence rates of various types of cirAEs across distinct classes of ICI-based therapies need to be elucidated. In this study, we address cirAE incidence in these novel ICI-based regimens and further examine cirAEs among several tumor types to broaden what is known about these common adverse effects. Further, the impact of cirAE incidence on survival, response, and duration of therapy has not been robustly examined across ICI-based regimens. In this meta-analysis, at the study level, we examine the incidence and prognostic associations of cirAEs across solid tumors and multiple ICI regimens, including monotherapy and combination with anti-angiogenic agents, chemotherapy, or other ICIs.

## 2. Materials and Methods

### 2.1. Study Identification

We searched PubMed, Embase, and ClinicalTrials.gov (accessed on 15 October 2023) to identify clinical trials with immune checkpoint inhibitors to include in this analysis.

PubMed was searched for clinical trials using the following terms: “atezolizumab”, “avelumab”, “cemiplimab”, “dostarlimab”, “durvalumab”, “ipilimumab”, “nivolumab”, “pembrolizumab”, “melanoma”, “carcinoma, transitional cell”, “bladder cancer”, “urothelial carcinoma”, “carcinoma, non-small-cell lung”, “non small lung carcinoma”, “non small lung cancer”, “carcinoma, renal cell” and “renal cell carcinoma.” Results were filtered using the criteria of human studies in the English language to identify 1848 studies that spanned from 2005 to 2022. Similar search terms were used for ClinicalTrials.gov (yielding 281 results) and Embase (yielding 2471 results).

All queries were performed on 4th April 2022. Duplicate studies were removed prior to the screening of a total of 3860 studies for inclusion or exclusion utilizing Covidence systematic review software (Veritas Health Innovation, Melbourne, Australia. Available at www.covidence.org, accessed on 15 October 2023).

### 2.2. Study Selection

Studies were included if they (1) reported incidence of cutaneous treatment-related and/or irAEs; (2) enrolled at least 30 patients and reported cirAEs within at least one study cohort which enrolled a minimum of 30 patients; (3) were prospective clinical trials in adult patients with either melanoma, renal cell carcinoma, urothelial carcinoma, or non-small cell lung cancer; and (4) involved ICIs given intravenously either alone or in combination with another ICI, chemotherapy, or antiangiogenic therapies (including bevacizumab, lenvatinib, axitinib, sunitinib, or cabozantinib). We included only the above cancer types to allow for comparison across cancer types.

Studies were excluded if they (1) involved ICIs in combination with radiotherapy or an agent not listed in the inclusion criteria; (2) involved neoadjuvant ICIs; (3) reported pooled data from multiple trials; or (4) reported on a study subgroup and had the entire trial data reported elsewhere. When multiple publications reported on the same trial, the manuscript with the longer follow-up time was selected unless cirAEs were not reported, in which case the article with the next longest available follow-up with data on cirAEs was selected.

**Figure 1:**
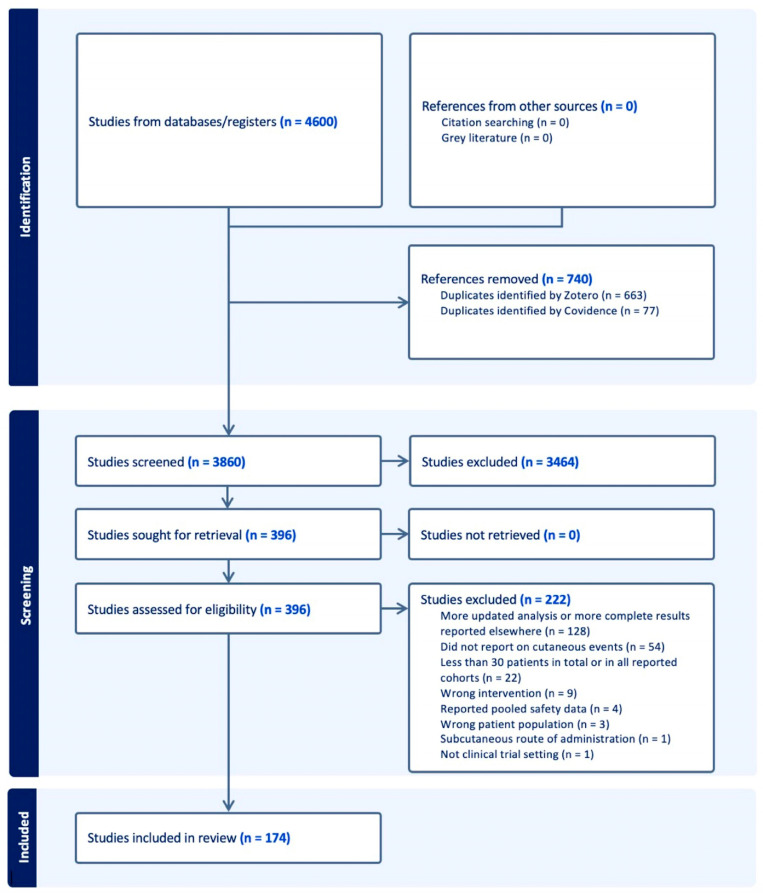
PRISMA flowchart of included studies.

A total of 396 studies were included in the full-text review following screening of the initial title and abstract. Following full-text review, 174 studies were included (Figure 1). Two reviewers reviewed studies during title and abstract screening, and one reviewer extracted data from the included studies. A second reviewer was available to review select full-text articles to determine the additional exclusion of studies during the full-text review. The systematic review followed the recommendations of the Preferred Reporting Items for Systematic Reviews and Meta-Analyses (PRISMA). The protocol has not been registered.

### 2.3. Data Extraction

The data collected included trial phase, tumor type, treatment, treatment class (e.g., anti-PD-1 or anti-VEGF), dose of anti-CTLA-4 agents (in mg/kg) when applicable, ICI treatment duration (median months), objective response rate (ORR), duration of response (DOR, median months), overall survival (OS, median months), progression-free survival (PFS, median months), and follow-up length (median months) when available. If the duration of treatment was recorded according to doses of the treatment, the duration of ICI treatment was estimated in months utilizing the reported dose frequency and schedule. In studies including patients with brain metastases and reporting both intracranial and extracranial ORR, intracranial ORR was collected.

Data on cirAEs were recorded according to treatment-related adverse events within the text or Supplementary Materials. The overall number of patients with an unspecified cirAE (all grade, grade 3–4, and grade 5) was collected when available. Additionally, commonly reported categories of cirAEs, including pruritis, vitiligo, rash, and maculopapular rash (all grade and grade 3–4), were recorded when available. Other specific cirAEs were noted, such as erythema multiforme, pemphigoid, psoriasis, severe cutaneous reaction, or stomatitis, but were not formally analyzed given their low numbers.

### 2.4. Statistical Methods

We estimated the overall incidence of adverse effects across studies, including grouped cirAEs, rash, pruritis, and vitiligo, using meta-analysis. The random-effects model was employed to provide an estimate of the overall effect while accounting for potential between-study variability (heterogeneity was assessed using Cochran’s Q test and I^2^ statistic) [15,16]. The incidence of cirAEs by subgroup of cancer type, class of drug, and dose of ipilimumab was evaluated via random-effects models for grouped cirAEs, rash, and pruritis. Due to the smaller sample sizes of studies reporting vitiligo, which were predominantly studies on melanoma given that vitiligo is a cirAE more commonly associated with melanoma, a fixed-effect model was utilized for estimates of vitiligo incidence. Associations between cirAEs and outcome measures of ORR, OS, PFS, and DOR were estimated in separate meta regression models, adjusted according to the phase of the trial (early: phase 1/2 vs. late: phase 3/4), treatment type, and tumor type. A *p*-value of <0.05 was considered statistically significant. 95% confidence intervals were provided for all point estimates. All statistical analyses were performed using R version 4.2.1. Due to the nature of the prediction model, some of the predicted values fell outside the [0, 1] range. The corresponding bubble plot figures have been cropped to exclude these unrealistic values.

## 3. Results

### 3.1. Overall Incidence of cirAEs among All Studies

A total of 396 studies were retrieved for full-text review following initial screening of the title and abstract. Based on inclusion criteria, 174 studies were included, comprising 219 cohorts with a total of 46,134 patients [17,18,19,20,21,22,23,24,25,26,27,28,29,30,31,32,33,34,35,36,37,38,39,40,41,42,43,44,45,46,47,48,49,50,51,52,53,54,55,56,57,58,59,60,61,62,63,64,65,66,67,68,69,70,71,72,73,74,75,76,77,78,79,80,81,82,83,84,85,86,87,88,89,90,91,92,93,94,95,96,97,98,99,100,101,102,103,104,105,106,107,108,109,110,111,112,113,114,115,116,117,118,119,120,121,122,123,124,125,126,127,128,129,130,131,132,133,134,135,136,137,138,139,140,141,142,143,144,145,146,147,148,149,150,151,152,153,154,155,156,157,158,159,160,161,162,163,164,165,166,167,168,169,170,171,172,173,174,175,176,177,178,179,180,181,182,183,184,185,186,187,188,189,190]. We examined the incidence of pruritis, vitiligo, and rash across all studies reporting these cirAEs. In total, 131 studies (178 cohorts, 38,736 patients) focused on pruritis; the incidence was estimated as 18.0% (95% CI 16.4–19.7) by random-effects modeling (Supplementary Figure S1). The estimated incidences of vitiligo and rash from 31 studies, largely studies on melanoma reporting on vitiligo (40 cohorts, 7693 patients), and 142 studies reporting on rash (190 cohorts, 42,332 patients) were 6.6% (95% CI 6.0–7.2) and 16.7% (95% CI 15.1–18.4), respectively (Supplementary Figures S2 and S3). We also evaluated the incidence of cirAEs across all studies reporting grouped cirAEs, comprising 65 cohorts from 45 studies and 15,850 patients. The overall incidence of all-grade cirAEs was 34.8% (95% CI 30.6–39.1), as estimated by random-effects modeling (Supplementary Figure S4).

### 3.2. Overall Incidence of cirAEs by Drug Class

Since the incidence of cirAEs likely varies by drug class, we grouped studies into the following regimens: PD-1 monotherapy; PD-L1 monotherapy; immunotherapy (IO) combination therapy (CTLA-4 in combination with either PD-1 or PD-L1 agents); IO + antiangiogenic agents; IO + chemotherapy (chemotherapy with any ICI agent with or without an antiangiogenic drug); and CTLA-4 monotherapy.

**Figure 2:**
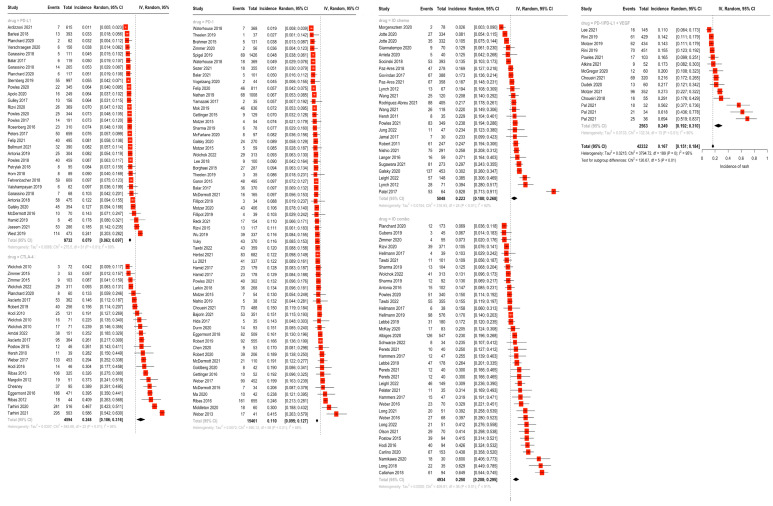
Overall incidence of rash by drug class groupings via random-effects modeling of 142 studies reporting rash. Highest incidence of rash was seen for IO combination (25.1%) followed by IO + antiangiogenic combination (24.9%), CTLA-4 monotherapy (24.8%), and IO + chemotherapy (22.3%). Rash was less frequent in anti-PD-1 (11.0%) and PD-L1 monotherapy (7.9%) [17,18,19,20,21,22,23,24,25,26,27,28,29,30,31,32,33,34,35,36,37,38,39,40,41,42,43,44,45,46,47,48,49,50,51,52,53,54,55,56,57,58,59,60,61,62,63,64,65,66,67,68,69,70,71,72,73,74,75,76,77,78,79,80,81,82,83,84,85,86,87,88,89,90,91,92,93,94,95,96,97,98,99,100,101,102,103,104,105,106,107,108,109,110,111,112,113,114,115,116,117,118,119,120,121,122,123,124,125,126,127,128,129,130,131,132,133,134,135,136,137,138,139,140,141,142,143,144,145,146,147,148,149,150,151,152,153,154,155,156,157,158,159,160,161,162,163,164,165,166,167,168,169,170,171,172,173,174,175,176,177,178,179,180,181,182,183,184,185,186,187,188,189,190].

**Figure 3:**
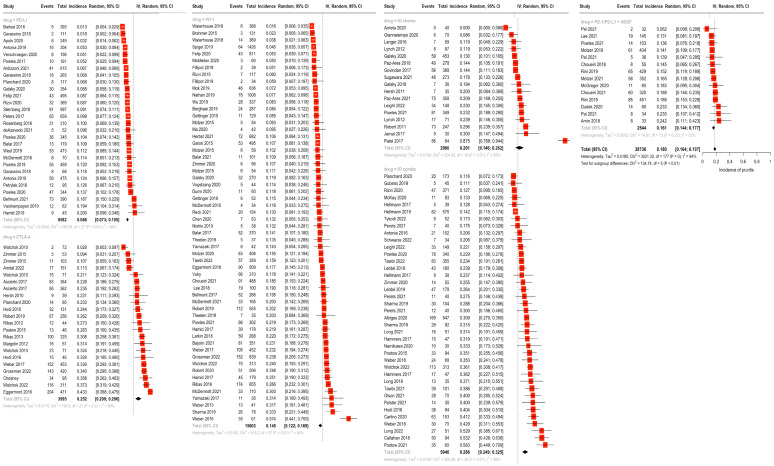
Overall incidence of pruritis by drug class groupings via random-effects modeling of 131 studies reporting pruritis. Highest incidence of pruritis was seen in the IO combination group at 28.6%, followed by CTLA-4 monotherapy (25.2%), IO + chemotherapy (20.1%), IO + antiangiogenic (16.1%), PD-1 monotherapy (14.5%), and PD-L1 monotherapy (8.8%) [17,18,19,20,21,22,23,24,25,26,27,28,29,30,31,32,33,34,35,36,37,38,39,40,41,42,43,44,45,46,47,48,49,50,51,52,53,54,55,56,57,58,59,60,61,62,63,64,65,66,67,68,69,70,71,72,73,74,75,76,77,78,79,80,81,82,83,84,85,86,87,88,89,90,91,92,93,94,95,96,97,98,99,100,101,102,103,104,105,106,107,108,109,110,111,112,113,114,115,116,117,118,119,120,121,122,123,124,125,126,127,128,129,130,131,132,133,134,135,136,137,138,139,140,141,142,143,144,145,146,147,148,149,150,151,152,153,154,155,156,157,158,159,160,161,162,163,164,165,166,167,168,169,170,171,172,173,174,175,176,177,178,179,180,181,182,183,184,185,186,187,188,189,190].

**Figure 4:**
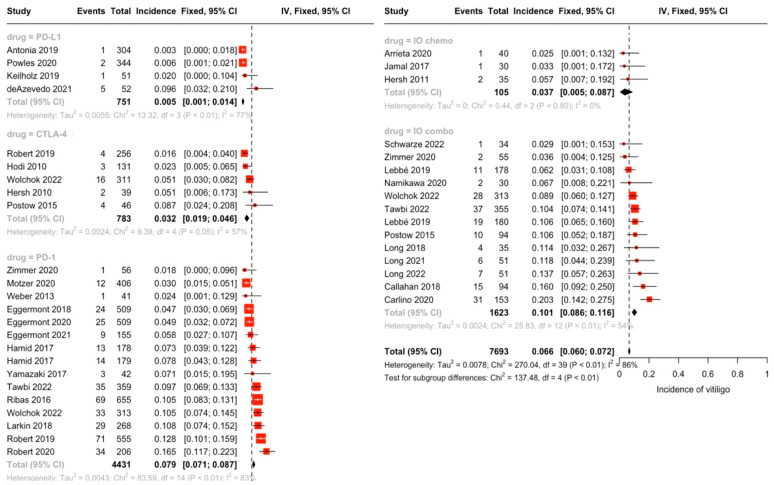
Overall incidence of vitiligo by drug class groupings via fixed-effect modeling of 31 studies reporting vitiligo. Incidence of vitiligo was highest in the IO combination (10.1%) and PD-1 monotherapy (7.9%) groups, followed by the IO + chemotherapy (3.7%), CTLA-4 monotherapy (3.2%), and PD-L1 monotherapy (0.54%) groups. No studies on IO + antiangiogenic combination regimens reported vitiligo [17,18,19,20,21,22,23,24,25,26,27,28,29,30,31,32,33,34,35,36,37,38,39,40,41,42,43,44,45,46,47,48,49,50,51,52,53,54,55,56,57,58,59,60,61,62,63,64,65,66,67,68,69,70,71,72,73,74,75,76,77,78,79,80,81,82,83,84,85,86,87,88,89,90,91,92,93,94,95,96,97,98,99,100,101,102,103,104,105,106,107,108,109,110,111,112,113,114,115,116,117,118,119,120,121,122,123,124,125,126,127,128,129,130,131,132,133,134,135,136,137,138,139,140,141,142,143,144,145,146,147,148,149,150,151,152,153,154,155,156,157,158,159,160,161,162,163,164,165,166,167,168,169,170,171,172,173,174,175,176,177,178,179,180,181,182,183,184,185,186,187,188,189,190].

In random-effects modeling of studies reporting rash (142 studies), the highest incidence of rash was seen for the IO combination (25.1%), followed by the IO + antiangiogenic combination (24.9%), CTLA-4 monotherapy (24.8%), and IO + chemotherapy (22.3%). Rash was less frequent in anti-PD-1 (11.0%) and PD-L1 monotherapy (7.9%) (Figure 2). Similarly, the incidence of pruritis (reported by 131 studies) was highest in the IO combination group at 28.6%, followed by CTLA-4 monotherapy (25.2%), IO + chemotherapy (20.1%), IO + antiangiogenic (16.1%), PD-1 monotherapy (14.5%) and PD-L1 monotherapy (8.8%) (Figure 3). There were statistically significant differences between subgroups for both rash and pruritis (*p* < 0.001). A fixed-effect model was used to examine incidence of vitiligo between drug subgroups due to the smaller sample size per grouping (31 studies total). No studies on IO + antiangiogenic combination regimens reported vitiligo, possibly due to the infrequent use of these agents in melanoma cases, where vitiligo is more commonly encountered. The incidence of vitiligo, as estimated by the fixed-effect model, was highest for the IO combination (10.1%) and PD-1 monotherapy (7.9%), followed by the IO + chemotherapy (3.7%), CTLA-4 monotherapy (3.2%), and PD-L1 monotherapy (0.54%) groups (*p* < 0.001) (Figure 4).

Through random-effects modeling, the highest incidence of all-grade cirAEs, analyzed in studies reporting cirAEs, was seen in the IO combination group at 48.9% and IO + chemotherapy at 44.8%, although a single IO + anti-angiogenic cohort showed very high rates of cirAEs (78.8%). The overall incidence of all-grade cirAEs for ICI monotherapy regimens was higher for CTLA-4 monotherapy (40.6%) compared with PD-1 (22.4%) or PD-L1 (16.9%) monotherapy (Supplementary Figure S5). Differences between subgroups were statistically significant (*p* < 0.0001).

### 3.3. Overall Incidence of cirAEs by Cancer Type

The incidences of rash, pruritis, and vitiligo were highest in melanoma cases. The incidence of rash was 12.4% for NSCLC, 10.5% for urothelial carcinoma, 25.3% for melanoma, and 17.7% for RCC (*p* < 0.001). The incidence of pruritis was 10.4% for NSCLC, 14.9% for urothelial carcinoma, 28.2% for melanoma, and 16.8% for RCC (*p* < 0.001). The incidence of vitiligo by cancer type was assessed via a fixed-effect model, as very few studies outside of those on melanoma reported vitiligo. The incidence of vitiligo was 7.9% in melanoma and ranged from 0.2 to 3% in the remaining cancer groups (*p* < 0.001).

Melanoma, urothelial carcinoma, and renal cell carcinoma were found to have similar incidences of all-grade cirAEs by random-effects modeling for studies reporting grouped cirAEs: 40.1%, 42.1%, and 41.3%, respectively. All-grade cirAEs were lower in non-small-cell lung cancer, at 20.8% (*p* < 0.001 for between-group differences).

### 3.4. cirAEs and Dose of CTLA-4 Regimens

To determine whether anti-CTLA-4-associated cirAEs were dose-related, we assessed the incidence of adverse events according to the ipilimumab monotherapy dose (1 mg/kg and under, 3 mg/kg, and 10 mg/kg) and the ipilimumab dose in combination with anti-PD-1 agents (1 mg/kg and 3 mg/kg).

The incidence of pruritis was highest in the ipilimumab 3 mg/kg + anti-PD-1 group at 34.8%, followed by the ipilimumab 10 mg/kg (32.9%), ipilimumab 1 mg/kg + anti-PD-1 (25.9%), ipilimumab 3 mg/kg (23.0%), and ipilimumab 1 mg/kg or under groups (2.8%) (Supplementary Figure S6). Similarly, the incidence of rash was highest in the ipilimumab 10 mg/kg group at 35.5%, followed by the ipilimumab 3 mg/kg + anti-PD-1 (30.5%), ipilimumab 1 mg/kg + anti-PD-1 (21.7%), ipilimumab 3 mg/kg (21.3%), and ipilimumab 1 mg/kg or under groups (4.2%) (Supplementary Figure S7). Studies reporting vitiligo only encompassed three dosing regimens; the incidence of vitiligo was highest for ipilimumab 1 mg/kg + anti-PD-1 at 14.7%, followed by the ipilimumab 3 mg/kg + anti-PD-1 (8.5%) and ipilimumab 3 mg/kg (3.2%) groups. Tests for differences between subgroups for each adverse event type were significant (*p* < 0.001) (Supplementary Figure S8). The incidence of overall cirAEs by random-effects modeling was highest in the ipilimumab 3 mg/kg + anti-PD-1 group at 52.6%, followed by ipilimumab 10 mg/kg (48.5%), ipilimumab 1 mg/kg + anti-PD-1 (45.7%), ipilimumab 3 mg/kg (37.4%), and ipilimumab 1 mg/kg or under (12.5%) (Supplementary Figure S9). Overall, cirAE incidence appeared to be dose-related when ipilimumab was used as a monotherapy, with less clear trends in the combination regimens.

### 3.5. Treatment Duration and cirAEs

**Figure 5:**
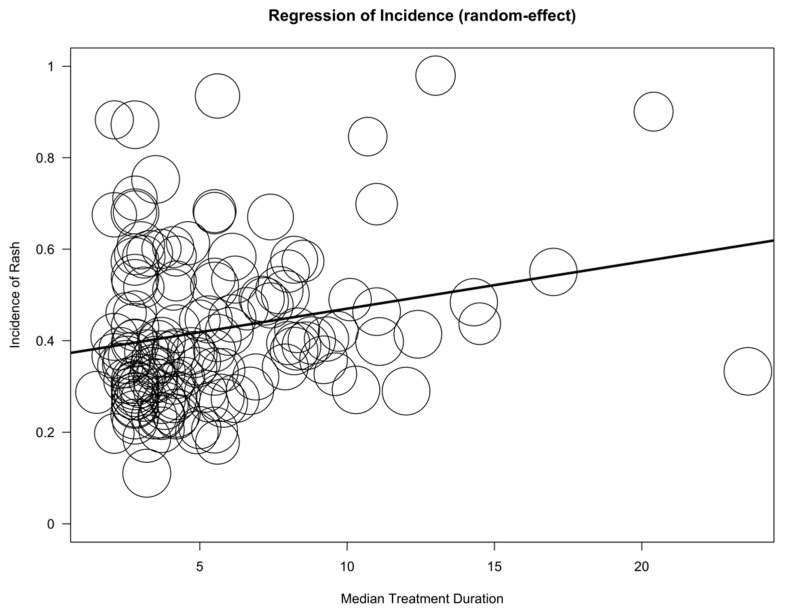
Bubble plot demonstrating the estimated regression slope for incidence of rash and duration of treatment (median months). Rash and median treatment duration were positively correlated, with an expected rise in incidence of 0.01% per month of treatment duration (*p* = 0.0116).

To assess whether cirAEs were associated with the duration of treatment, we fitted meta-regression models to adverse event incidence with median treatment duration, adjusting for tumor type, treatment class, and phase of trial. While the median treatment duration was not found to be associated with pruritus or vitiligo, it was positively associated with rash (*p* = 0.0116) (Figure 5 and Supplementary Figure S10). Additionally, the later phases of the trials on melanoma (compared to NSCLC, RCC, or urothelial carcinoma) were associated with increased cirAE incidence, while the treatment class (specifically PD-1 and PD-L1 blockade) was associated with lower cirAE incidence.

### 3.6. Outcome Measures and cirAEs; Response Rate, Duration of Response, Progression-Free Survival, and Overall Survival

**Figure 6:**
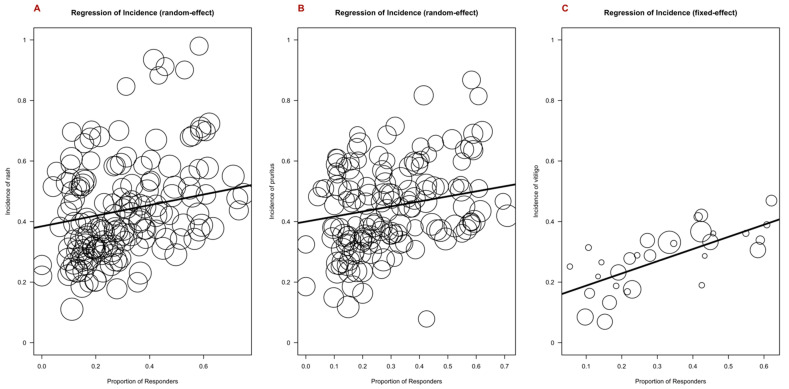
Bubble plot demonstrating the estimated regression slope for incidence of rash (**A**), pruritis (**B**), and vitiligo (**C**), as well as response rate. Response rate was positively correlated with the incidence of pruritis, vitiligo, and rash, with an expected rise in incidence of 0.17% (*p* = 0.0238) for pruritis, 0.40% (*p* = 0.0010) for vitiligo, and 0.18% (*p* = 0.0413) for rash per percentage increase in response rate.

**Figure 7:**
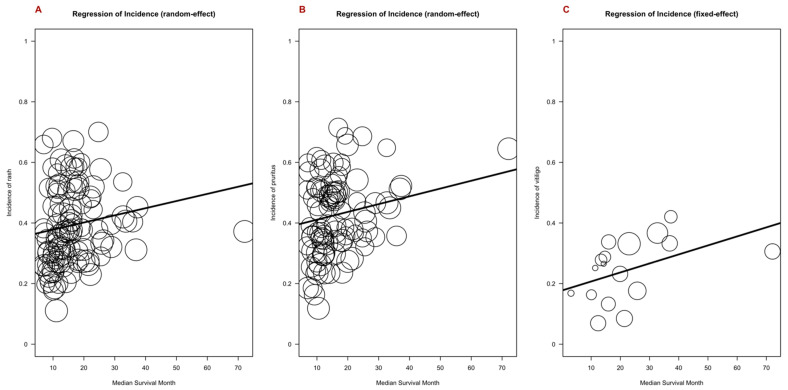
Bubble plot demonstrating the estimated regression slope for incidence of rash (**A**), pruritis (**B**), and vitiligo (**C**), as well as overall survival. Overall survival was positively correlated with the incidence of pruritis, vitiligo, and rash, and this association was significant for vitiligo (*p* = 0.0483), with an expected rise in incidence of vitiligo of 0.003% per additional month of overall survival.

Given the previously published link between cutaneous and other irAEs and improved outcomes at an individual patient level, we performed a meta-regression analysis of adverse event incidence for rash, pruritis, and vitiligo, including clinical outcome measures. Covariates included trial phase, drug class, and tumor type. The response rate was positively correlated with the incidence of pruritis (*p* = 0.0238), vitiligo (*p* = 0.0010), and rash (*p* = 0.0413) (Figure 6). The duration of the response was positively correlated with the incidence of rash, pruritis, and vitiligo, although these associations were not statistically significant (Supplementary Figure S11). Overall survival was positively associated with the incidence of vitiligo (*p* = 0.0483) (Figure 7). Progression-free survival was positively associated with the incidence of pruritis (*p* = 0.0207) and rash (*p* = 0.0351), whereas a negative association was found with vitiligo (*p* = 0.0029) (Supplementary Figure S12).

## 4. Discussion

In our analysis, we provide the most comprehensive meta-analysis of cirAEs by type, therapy regimen, and tumor, and we correlate cirAEs with outcomes across clinical trials. Across studies, cirAEs were more frequent with regimens containing combinations of ICIs and in melanoma compared to other tumor types, highlighting the impact of new and more frequently used combination regimens, such as that of ICI and anti-angiogenic drugs or dual checkpoint blockade with CTLA-4 agents. We observed general associations between the incidence of cirAEs and improved treatment outcomes, as well as with treatment duration, demonstrating the potential impact of cirAE development as a prognostic tool for mapping therapeutic outcomes and the risk of late-onset cirAEs.

Our analysis provides a benchmark for the incidence of cirAEs with distinct ICI-based therapeutic classes, and demonstrates the variability of cirAEs between regimens. Treatment regimens with combined ICIs, as well as CTLA-4-containing regimens, were consistently associated with higher incidence of all cirAEs analyzed, while PD-1/PD-L1 monotherapy regimens were associated with lower incidence. ICIs in combination with antiangiogenic agents were associated with higher rates of rash than pruritis, while no studies using this regimen reported vitiligo. This may reflect mechanistic differences by which varying agents induce differing cirAEs, and in some cases, differences in the settings in which new regimens are used. For example, combination PD-1/CTLA-4 blockade may appear to produce more vitiligo, but this may be due to its more frequent use in melanoma, whereas anti-angiogenic agents are not used in melanoma. On the other hand, cirAEs may appear more frequently in one tumor type (e.g., melanoma) due to the more frequent use (and at higher doses) of CTLA-4 blockade in that tumor type.

This analysis also highlights the association between cirAE types and therapeutic outcomes not previously reported by other meta-analyses [12,13,14]. A prior meta-analysis examining 15 studies of ICI monotherapy did not find significant associations between rash, pruritis, and overall survival or progression free survival, although it did not include vitiligo or combination ICI regimens in the study [12]. In our analysis, rash, pruritis, and vitiligo were significantly associated with response rate and PFS, as was vitiligo and overall survival. Despite observing significant associations between vitiligo and improved response rate and overall survival, we found vitiligo to be negatively associated with PFS, which may be due to the small sample size and 1–2 outlier studies. All other outcome measures, including response rate, duration of response, and overall survival, demonstrated generally positive correlations with each type of cirAE. Rash also was associated with the duration of therapy, highlighting the risk of later-onset cirAEs [191,192].

Limitations to our analysis are important to consider. Subtypes of cirAEs were reported at various levels of detail across studies, with determination of whether more specific adverse events were to be reported as part of broad groupings (e.g., rash and pruritis) left up to the investigators in each study. This may contribute to significant variability between studies and, thus, the accuracy of the results of this study. While most studies reported adverse events based upon the CTCAE, no criteria specific to more nuanced cirAE reporting exists, and the involvement of dermatologists in the reporting of cirAE likely varied across studies. Furthermore, we examined all data at the study level and not at the individual patient level, which also constitutes a source of bias in this analysis.

While meta-regression can offer valuable insights into the relationship between study characteristics and effect sizes in meta-analysis, due to the aggregated nature of meta-analysis, associations detected at the study level may not accurately reflect individual-level relationships. The interpretation of findings from study-level associations for individual studies should be carried out cautiously. Additionally, meta-regression assumes linearity in the relationship between study characteristics and effect sizes. However, this assumption may not hold in all cases, and the relationship could be nonlinear or exhibit threshold effects. In our analysis, we opted to use a linear model like meta-regression so as not to reduce the power of the analysis. Nonetheless, the assumption of linearity is an important consideration when interpreting the results of this meta-analysis.

Our analysis validates the associations of cirAEs with clinical outcomes at a study level across a range of solid tumors and ICI monotherapy and combination regimens, and provides benchmark incidence rates across these regimens. As the use of ICIs in a variety of therapeutic combinations and tumors increases, future studies capturing more detail regarding specific cirAEs and patient-level data may allow for more specific prognostic associations to be drawn and inform the care of individual patients. Ultimately, further understanding of the mechanisms by which cirAEs influence therapeutic response may assist oncologists and dermatologists in the management of both diseases and adverse events, as well as patient-specific counseling regarding prognosis.

## 5. Conclusions

Our analysis provides a benchmark for the incidence of cirAEs with distinct ICI-based therapeutic classes. It demonstrates the variability of cirAE incidence between regimens, as well as the cirAE type and associated therapeutic benefit. We found that treatment regimens with combined ICIs, as well as CTLA-4-containing regimens, were consistently associated with higher incidence of all cirAEs analyzed, while PD-1/PD-L1 monotherapy regimens were associated with lower incidence. In our analysis, response rate and PFS were significantly associated with increased rash, pruritis, and vitiligo incidence. Positive correlations were seen between increased cirAE incidence of all types, duration of response, and overall survival, though this was significant only for vitiligo and overall survival.

## Supplementary Materials

The following supporting information can be downloaded at: https://www.mdpi.com/article/10.3390/cancers16020340/s1. Figure S1. Overall incidence of pruritis across studies; Figure S2. Overall incidence of rash across studies; Figure S3. Overall incidence of vitiligo across studies; Figure S4. Overall incidence of cirAEs across studies; Figure S5. Overall incidence of cirAEs by drug class groupings; Figure S6. Overall incidence of pruritis by ipilimumab dose groupings; Figure S7. Overall incidence of rash by ipilimumab dose groupings; Figure S8. Overall incidence of vitiligo by ipilimumab dose groupings; Figure S9. Overall incidence of cirAEs by ipilimumab dose groupings; Figure S10: Bubble plots demonstrating the estimated regression slope for incidence of pruritis (A) and vitiligo (B) and duration of treatment (median months); Figure S11: Bubble plot demonstrating the estimated regression slope for incidence of rash (A), pruritis (B), and vitiligo (C) and duration of response; Figure S12: Bubble plot demonstrating the estimated regression slope for incidence of rash (A), pruritis (B), and vitiligo (C) and progression-free survival.

## Abbreviations

PD-1/PD-L1	programmed death-1/ligand-1
CTLA-4	cytotoxic T lymphocyte antigen-4
ICIs	Immune checkpoint inhibitors
irAEs	immune-related adverse events
cirAEs	cutaneous immune-related adverse events
VEGF	vascular endothelial growth factor
ORR	objective response rate
DOR	duration of response
OS	overall survival
PFS	progression free survival
IO	Immunotherapy
CTCAE	Common Terminology Criteria for Adverse Events

## References

[1] Johnson D.B., Jakubovic B.D., Sibaud V., Sise M.E. (2020). Balancing Cancer Immunotherapy Efficacy and Toxicity. J. Allergy Clin. Immunol. Pract..

[2] Quach H.T., Johnson D.B., LeBoeuf N.R., Zwerner J.P., Dewan A.K. (2021). Cutaneous adverse events caused by immune checkpoint inhibitors. J. Am. Acad. Dermatol..

[3] Cosio T., Coniglione F., Flaminio V., Gaziano R., Coletta D., Petruccelli R., Dika E., Bianchi L., Campione E. (2023). Pyodermitis during Nivolumab Treatment for Non-Small Cell Lung Cancer: A Case Report and Review of the Literature. Int. J. Mol. Sci..

[4] Zhang S., Tang K., Wan G., Nguyen N., Lu C., Ugwu-Dike P., Raval N., Seo J., Alexander N.A., Jairath R. (2023). Cutaneous immune-related adverse events are associated with longer overall survival in advanced cancer patients on immune checkpoint inhibitors: A multi-institutional cohort study. J. Am. Acad. Dermatol..

[5] Tang K., Seo J., Tiu B.C., Le T.K., Pahalyants V., Raval N.S., Ugwu-Dike P.O., Zubiri L., Naranbhai V., Carrington M. (2022). Association of Cutaneous Immune-Related Adverse Events With Increased Survival in Patients Treated With Anti–Programmed Cell Death 1 and Anti–Programmed Cell Death Ligand 1 Therapy. JAMA Dermatol..

[6] Quach H.T., Dewan A.K., Davis E.J., Ancell K.K., Fan R., Ye F., Johnson D.B. (2019). Association of Anti–Programmed Cell Death 1 Cutaneous Toxic Effects With Outcomes in Patients With Advanced Melanoma. JAMA Oncol..

[7] Bottlaender L., Amini-Adle M., Maucort-Boulch D., Robinson P., Thomas L., Dalle S. (2020). Cutaneous adverse events: A predictor of tumour response under anti-PD-1 therapy for metastatic melanoma, a cohort analysis of 189 patients. J. Eur. Acad. Dermatol. Venereol..

[8] Berner F., Bomze D., Diem S., Ali O.H., Fässler M., Ring S., Niederer R., Ackermann C.J., Baumgaertner P., Pikor N. (2019). Association of Checkpoint Inhibitor–Induced Toxic Effects With Shared Cancer and Tissue Antigens in Non–Small Cell Lung Cancer. JAMA Oncol..

[9] Larsabal M., Marti A., Jacquemin C., Rambert J., Thiolat D., Dousset L., Taieb A., Dutriaux C., Prey S., Boniface K. (2017). Vitiligo-like lesions occurring in patients receiving anti-programmed cell death-1 therapies are clinically and biologically distinct from vitiligo. J. Am. Acad. Dermatol..

[10] Ferro M., Crocetto F., Tataru S., Barone B., Dolce P., Lucarelli G., Sonpavde G., Musi G., Antonelli A., Veccia A. (2023). Predictors of Efficacy of Immune Checkpoint Inhibitors in Patients With Advanced Urothelial Carcinoma: A Systematic Review and Meta-Analysis. Clin. Genitourin. Cancer.

[11] Basudan A.M. (2022). The Role of Immune Checkpoint Inhibitors in Cancer Therapy. Clin. Pract..

[12] Han Y., Wang J., Xu B. (2021). Cutaneous adverse events associated with immune checkpoint blockade: A systematic review and meta-analysis. Crit. Rev. Oncol. Hematol..

[13] Abdel-Rahman O., ElHalawani H., Fouad M. (2015). Risk of cutaneous toxicities in patients with solid tumors treated with immune checkpoint inhibitors: A meta-analysis. Future Oncol..

[14] Mineiro dos Santos Garrett N.F., Carvalho da Costa A.C., Barros Ferreira E., Damiani G., Diniz dos Reis P.E., Inocêncio Vasques C. (2021). Prevalence of dermatological toxicities in patients with melanoma undergoing immunotherapy: Systematic review and meta-analysis. PLoS ONE.

[15] Cochran W.G. (1950). The Comparison of Percentages in Matched Samples. Biometrika.

[16] Higgins J.P.T., Thompson S.G. (2002). Quantifying heterogeneity in a meta-analysis. Stat. Med..

[17] Durm G.A., Jabbour S.K., Althouse S.K., Liu Z., Sadiq A.A., Zon R.T., Jalal S.I., Kloecker G.H., Williamson M.J., Reckamp K.L. (2020). A phase 2 trial of consolidation pembrolizumab following concurrent chemoradiation for patients with unresectable stage III non-small cell lung cancer: Hoosier Cancer Research Network LUN 14-179. Cancer.

[18] Hersh E.M., O’Day S.J., Powderly J., Khan K.D., Pavlick A.C., Cranmer L.D., Samlowski W.E., Nichol G.M., Yellin M.J., Weber J.S. (2011). A phase II multicenter study of ipilimumab with or without dacarbazine in chemotherapy-naive patients with advanced melanoma. Investig. New Drugs.

[19] Patel S.P., Kim D.W., Bassett R.L., Cain S., Washington E., Hwu W.-J., Kim K.B., Papadopoulos N.E., Homsi J., Hwu P. (2017). A phase II study of ipilimumab plus temozolomide in patients with metastatic melanoma. Cancer Immunol. Immunother. CII.

[20] Hamid O., Schmidt H., Nissan A., Ridolfi L., Aamdal S., Hansson J., Guida M., Hyams D.M., Gomez H., Bastholt L. (2011). A prospective phase II trial exploring the association between tumor microenvironment biomarkers and clinical activity of ipilimumab in advanced melanoma. J. Transl. Med..

[21] Hayashi H., Sugawara S., Fukuda Y., Fujimoto D., Miura S., Ota K., Ozawa Y., Hara S., Tanizaki J., Azuma K. (2022). A Randomized Phase II Study Comparing Nivolumab with Carboplatin-Pemetrexed for EGFR-Mutated NSCLC with Resistance to EGFR Tyrosine Kinase Inhibitors (WJOG8515L). Clin. Cancer Res..

[22] Weber J., Thompson J.A., Hamid O., Minor D., Amin A., Ron I., Ridolfi R., Assi H., Maraveyas A., Berman D. (2009). A randomized, double-blind, placebo-controlled, phase II study comparing the tolerability and efficacy of ipilimumab administered with or without prophylactic budesonide in patients with unresectable stage III or IV melanoma. Clin. Cancer Res..

[23] Rizvi N.A., Mazieres J., Planchard D., Stinchcombe T.E., Dy G.K., Antonia S.J., Horn L., Lena H., Minenza E., Mennecier B. (2015). Activity and safety of nivolumab, an anti-PD-1 immune checkpoint inhibitor, for patients with advanced, refractory squamous non-small-cell lung cancer (CheckMate 063): A phase 2, single-arm trial. Lancet Oncol..

[24] Postow M.A., Goldman D.A., Shoushtari A.N., Betof Warner A., Callahan M.K., Momtaz P., Smithy J.W., Naito E., Cugliari M.K., Raber V. (2021). Adaptive Dosing of Nivolumab + Ipilimumab Immunotherapy Based Upon Early, Interim Radiographic Assessment in Advanced Melanoma (The ADAPT-IT Study). J. Clin. Oncol. Off. J. Am. Soc. Clin. Oncol..

[25] Felip E., Altorki N., Zhou C., Csoszi T., Vynnychenko I., Goloborodko O., Luft A., Akopov A., Martinez-Marti A., Kenmotsu H. (2021). Adjuvant atezolizumab after adjuvant chemotherapy in resected stage IB-IIIA non-small-cell lung cancer (IMpower010): A randomised, multicentre, open-label, phase 3 trial. Lancet.

[26] Bellmunt J., Hussain M., Gschwend J.E., Albers P., Oudard S., Castellano D., Daneshmand S., Nishiyama H., Majchrowicz M., Degaonkar V. (2021). Adjuvant atezolizumab versus observation in muscle-invasive urothelial carcinoma (IMvigor010): A multicentre, open-label, randomised, phase 3 trial. Lancet Oncol..

[27] Zimmer L., Livingstone E., Hassel J.C., Fluck M., Eigentler T., Loquai C., Haferkamp S., Gutzmer R., Meier F., Mohr P. (2020). Adjuvant nivolumab plus ipilimumab or nivolumab monotherapy versus placebo in patients with resected stage IV melanoma with no evidence of disease (IMMUNED): A randomised, double-blind, placebo-controlled, phase 2 trial. Lancet.

[28] Weber J., Mandala M., Del Vecchio M., Gogas H.J., Arance A.M., Cowey C.L., Dalle S., Schenker M., Chiarion-Sileni V., Marquez-Rodas I. (2017). Adjuvant nivolumab versus ipilimumab in resected stage III or IV melanoma. N. Engl. J. Med..

[29] Bajorin D.F., Witjes J.A., Gschwend J.E., Schenker M., Valderrama B.P., Tomita Y., Bamias A., Lebret T., Shariat S.F., Park S.H. (2021). Adjuvant Nivolumab versus Placebo in Muscle-Invasive Urothelial Carcinoma. N. Engl. J. Med..

[30] Choueiri T.K., Tomczak P., Park S.H., Venugopal B., Ferguson T., Chang Y.-H., Hajek J., Symeonides S.N., Lee J.L., Sarwar N. (2021). Adjuvant pembrolizumab after nephrectomy in renal-cell carcinoma. N. Engl. J. Med..

[31] Grossmann K.F., Othus M., Patel S.P., Tarhini A.A., Sondak V.K., Knopp M.V., Petrella T.M., Truong T.-G., Khushalani N.I., Cohen J.V. (2022). Adjuvant Pembrolizumab versus IFNalpha2b or Ipilimumab in Resected High-Risk Melanoma. Cancer Discov..

[32] Eggermont A.M.M., Blank C.U., Mandala M., Long G.V., Atkinson V., Dalle S., Haydon A., Lichinitser M., Khattak A., Carlino M.S. (2018). Adjuvant pembrolizumab versus placebo in resected stage III melanoma. N. Engl. J. Med..

[33] Planchard D., Reinmuth N., Orlov S., Fischer J.R., Sugawara S., Mandziuk S., Marquez-Medina D., Novello S., Takeda Y., Soo R. (2020). ARCTIC: Durvalumab with or without tremelimumab as third-line or later treatment of metastatic non-small-cell lung cancer. Ann. Oncol..

[34] Ribas A., Hamid O., Daud A., Hodi F.S., Wolchok J.D., Kefford R., Joshua A.M., Patnaik A., Hwu W.-J., Weber J.S. (2016). Association of Pembrolizumab With Tumor Response and Survival Among Patients With Advanced Melanoma. JAMA.

[35] Petrylak D.P., Powles T., Bellmunt J., Braiteh F., Loriot Y., Morales-Barrera R., Burris H.A., Kim J.W., Ding B., Kaiser C. (2018). Atezolizumab (MPDL3280A) Monotherapy for Patients With Metastatic Urothelial Cancer: Long-term Outcomes From a Phase 1 Study. JAMA Oncol..

[36] Balar A.V., Galsky M.D., Rosenberg J.E., Powles T., Petrylak D.P., Bellmunt J., Loriot Y., Necchi A., Hoffman-Censits J., Perez-Gracia J.L. (2017). Atezolizumab as first-line treatment in cisplatin-ineligible patients with locally advanced and metastatic urothelial carcinoma: A single-arm, multicentre, phase 2 trial. Lancet Lond. Engl..

[37] Socinski M.A., Jotte R.M., Cappuzzo F., Orlandi F., Stroyakovskiy D., Nogami N., Rodríguez-Abreu D., Moro-Sibilot D., Thomas C.A., Barlesi F. (2018). Atezolizumab for First-Line Treatment of Metastatic Nonsquamous NSCLC. N. Engl. J. Med..

[38] Jotte R., Cappuzzo F., Vynnychenko I., Stroyakovskiy D., Rodriguez-Abreu D., Hussein M., Soo R., Conter H.J., Kozuki T., Huang K.-C. (2020). Atezolizumab in Combination With Carboplatin and Nab-Paclitaxel in Advanced Squamous NSCLC (IMpower131): Results From a Randomized Phase III Trial. J. Thorac. Oncol..

[39] West H., McCleod M., Hussein M., Morabito A., Rittmeyer A., Conter H.J., Kopp H.-G., Daniel D., McCune S., Mekhail T. (2019). Atezolizumab in combination with carboplatin plus nab-paclitaxel chemotherapy compared with chemotherapy alone as first-line treatment for metastatic non-squamous non-small-cell lung cancer (IMpower130): A multicentre, randomised, open-label, phase 3 trial. Lancet Oncol..

[40] Rosenberg J.E., Hoffman-Censits J., Powles T., van der Heijden M.S., Balar A.V., Necchi A., Dawson N., O’Donnell P.H., Balmanoukian A., Loriot Y. (2016). Atezolizumab in patients with locally advanced and metastatic urothelial carcinoma who have progressed following treatment with platinum-based chemotherapy: A single-arm, multicentre, phase 2 trial. Lancet Lond. Engl..

[41] Rini B.I., Powles T., Atkins M.B., Escudier B., McDermott D.F., Suarez C., Bracarda S., Stadler W.M., Donskov F., Lee J.L. (2019). Atezolizumab plus bevacizumab versus sunitinib in patients with previously untreated metastatic renal cell carcinoma (IMmotion151): A multicentre, open-label, phase 3, randomised controlled trial. Lancet.

[42] Nishio M., Barlesi F., West H., Ball S., Bordoni R., Cobo M., Longeras P.D., Goldschmidt J., Novello S., Orlandi F. (2021). Atezolizumab Plus Chemotherapy for First-Line Treatment of Nonsquamous NSCLC: Results From the Randomized Phase 3 IMpower132 Trial. J. Thorac. Oncol..

[43] Powles T., Durán I., van der Heijden M.S., Loriot Y., Vogelzang N.J., De Giorgi U., Oudard S., Retz M.M., Castellano D., Bamias A. (2018). Atezolizumab versus chemotherapy in patients with platinum-treated locally advanced or metastatic urothelial carcinoma (IMvigor211): A multicentre, open-label, phase 3 randomised controlled trial. Lancet Lond. Engl..

[44] Fehrenbacher L., Spira A., Ballinger M., Kowanetz M., Vansteenkiste J., Mazieres J., Park K., Smith D., Artal-Cortes A., Lewanski C. (2016). Atezolizumab versus docetaxel for patients with previously treated non-small-cell lung cancer (POPLAR): A multicentre, open-label, phase 2 randomised controlled trial. Lancet.

[45] Galsky M.D., Arija J.Á.A., Bamias A., Davis I.D., De Santis M., Kikuchi E., Garcia-Del-Muro X., De Giorgi U., Mencinger M., Izumi K. (2020). Atezolizumab with or without chemotherapy in metastatic urothelial cancer (IMvigor130): A multicentre, randomised, placebo-controlled phase 3 trial. Lancet Lond. Engl..

[46] McDermott D.F., Sosman J.A., Sznol M., Massard C., Gordon M.S., Hamid O., Powderly J.D., Infante J.R., Fassò M., Wang Y.V. (2016). Atezolizumab, an Anti-Programmed Death-Ligand 1 Antibody, in Metastatic Renal Cell Carcinoma: Long-Term Safety, Clinical Activity, and Immune Correlates From a Phase Ia Study. J. Clin. Oncol. Off. J. Am. Soc. Clin. Oncol..

[47] Apolo A.B., Ellerton J.A., Infante J.R., Agrawal M., Gordon M.S., Aljumaily R., Gourdin T., Dirix L., Lee K.-W., Taylor M.H. (2020). Avelumab as second-line therapy for metastatic, platinum-treated urothelial carcinoma in the phase Ib JAVELIN Solid Tumor study: 2-year updated efficacy and safety analysis. J. Immunother. Cancer.

[48] Gulley J.L., Rajan A., Spigel D.R., Iannotti N., Chandler J., Wong D.J.L., Leach J., Edenfield W.J., Wang D., Grote H.J. (2017). Avelumab for patients with previously treated metastatic or recurrent non-small-cell lung cancer (JAVELIN Solid Tumor): Dose-expansion cohort of a multicentre, open-label, phase 1b trial. Lancet Oncol..

[49] Keilholz U., Mehnert J.M., Bauer S., Bourgeois H., Patel M.R., Gravenor D., Nemunaitis J.J., Taylor M.H., Wyrwicz L., Lee K.-W. (2019). Avelumab in patients with previously treated metastatic melanoma: Phase 1b results from the JAVELIN Solid Tumor trial. J. Immunother. Cancer.

[50] Powles T., Park S.H., Voog E., Caserta C., Valderrama B.P., Gurney H., Kalofonos H., Radulović S., Demey W., Ullén A. (2020). Avelumab Maintenance Therapy for Advanced or Metastatic Urothelial Carcinoma. N. Engl. J. Med..

[51] Vaishampayan U., Schöffski P., Ravaud A., Borel C., Peguero J., Chaves J., Morris J.C., Kotecki N., Smakal M., Zhou D. (2019). Avelumab monotherapy as first-line or second-line treatment in patients with metastatic renal cell carcinoma: Phase Ib results from the JAVELIN Solid Tumor trial. J. Immunother. Cancer.

[52] Motzer R.J., Penkov K., Haanen J., Rini B., Albiges L., Campbell M.T., Venugopal B., Kollmannsberger C., Negrier S., Uemura M. (2019). Avelumab plus Axitinib versus Sunitinib for Advanced Renal-Cell Carcinoma. N. Engl. J. Med..

[53] Barlesi F., Vansteenkiste J., Spigel D., Ishii H., Garassino M., de Marinis F., Ozguroglu M., Szczesna A., Polychronis A., Uslu R. (2018). Avelumab versus docetaxel in patients with platinum-treated advanced non-small-cell lung cancer (JAVELIN Lung 200): An open-label, randomised, phase 3 study. Lancet Oncol..

[54] Atkins M.B., Plimack E.R., Puzanov I., Fishman M.N., McDermott D.F., Cho D.C., Vaishampayan U., George S., Tarazi J.C., Duggan W. (2021). Axitinib plus pembrolizumab in patients with advanced renal-cell carcinoma: Long-term efficacy and safety from a phase Ib trial. Eur. J. Cancer.

[55] Pal S.K., McGregor B., Suárez C., Tsao C.-K., Kelly W., Vaishampayan U., Pagliaro L., Maughan B.L., Loriot Y., Castellano D. (2021). Cabozantinib in Combination With Atezolizumab for Advanced Renal Cell Carcinoma: Results From the COSMIC-021 Study. J. Clin. Oncol. Off. J. Am. Soc. Clin. Oncol..

[56] Langer C.J., Gadgeel S.M., Borghaei H., Papadimitrakopoulou V.A., Patnaik A., Powell S.F., Gentzler R.D., Martins R.G., Stevenson J.P., Jalal S.I. (2016). Carboplatin and pemetrexed with or without pembrolizumab for advanced, non-squamous non-small-cell lung cancer: A randomised, phase 2 cohort of the open-label KEYNOTE-021 study. Lancet Oncol..

[57] Leighl N.B., Laurie S.A., Goss G.D., Hughes B.G.M., Stockler M., Tsao M.-S., Hwang D.M., Joubert P., Kulkarni S., Blais N. (2022). CCTG BR34: A Randomized Phase 2 Trial of Durvalumab and Tremelimumab With or Without Platinum-Based Chemotherapy in Patients With Metastatic NSCLC. J. Thorac. Oncol..

[58] Sezer A., Kilickap S., Gumus M., Bondarenko I., Ozguroglu M., Gogishvili M., Turk H.M., Cicin I., Bentsion D., Gladkov O. (2021). Cemiplimab monotherapy for first-line treatment of advanced non-small-cell lung cancer with PD-L1 of at least 50%: A multicentre, open-label, global, phase 3, randomised, controlled trial. Lancet.

[59] Felip E., Ardizzoni A., Ciuleanu T., Cobo M., Laktionov K., Szilasi M., Califano R., Carcereny E., Griffiths R., Paz-Ares L. (2020). CheckMate 171: A phase 2 trial of nivolumab in patients with previously treated advanced squamous non-small cell lung cancer, including ECOG PS 2 and elderly populations. Eur. J. Cancer.

[60] Antonia S.J., Balmanoukian A., Brahmer J., Ou S.-H.I., Hellmann M.D., Kim S.-W., Ahn M.-J., Kim D.-W., Gutierrez M., Liu S.V. (2019). Clinical Activity, Tolerability, and Long-Term Follow-Up of Durvalumab in Patients With Advanced NSCLC. J. Thorac. Oncol. Off. Publ. Int. Assoc. Study Lung Cancer.

[61] Lam T.C., Tsang K.C., Choi H.C., Lee V.H., Lam K.O., Chiang C.L., So T.H., Chan W.W., Nyaw S.F., Lim F. (2021). Combination atezolizumab, bevacizumab, pemetrexed and carboplatin for metastatic EGFR mutated NSCLC after TKI failure. Lung Cancer.

[62] Long G.V., Atkinson V., Lo S., Sandhu S., Guminski A.D., Brown M.P., Wilmott J.S., Edwards J., Gonzalez M., Scolyer R.A. (2018). Combination nivolumab and ipilimumab or nivolumab alone in melanoma brain metastases: A multicentre randomised phase 2 study. Lancet Oncol..

[63] Hodi F.S., Chesney J., Pavlick A.C., Robert C., Grossmann K.F., McDermott D.F., Linette G.P., Meyer N., Giguere J.K., Agarwala S.S. (2016). Combined nivolumab and ipilimumab versus ipilimumab alone in patients with advanced melanoma: 2-year overall survival outcomes in a multicentre, randomised, controlled, phase 2 trial. Lancet Oncol..

[64] Jung H.A., Park S., Choi Y.-L., Lee S.-H., Ahn J.S., Ahn M.-J., Sun J.-M. (2022). Continuation of pembrolizumab with additional chemotherapy after progression with PD-1/PD-L1 inhibitor monotherapy in patients with advanced NSCLC. Clin. Cancer Res. Off. J. Am. Assoc. Cancer Res..

[65] Waterhouse D.M., Garon E.B., Chandler J., McCleod M., Hussein M., Jotte R., Horn L., Daniel D.B., Keogh G., Creelan B. (2020). Continuous Versus 1-Year Fixed-Duration Nivolumab in Previously Treated Advanced Non-Small-Cell Lung Cancer: CheckMate 153. J. Clin. Oncol. Off. J. Am. Soc. Clin. Oncol..

[66] Eggermont A.M., Meshcheryakov A., Atkinson V., Blank C.U., Mandala M., Long G.V., Barrow C., Di Giacomo A.M., Fisher R., Sandhu S. (2021). Crossover and rechallenge with pembrolizumab in recurrent patients from the EORTC 1325-MG/Keynote-054 phase III trial, pembrolizumab versus placebo after complete resection of high-risk stage III melanoma. Eur. J. Cancer.

[67] Yamazaki N., Kiyohara Y., Uhara H., Iizuka H., Uehara J., Otsuka F., Fujisawa Y., Takenouchi T., Isei T., Iwatsuki K. (2017). Cytokine biomarkers to predict antitumor responses to nivolumab suggested in a phase 2 study for advanced melanoma. Cancer Sci..

[68] Powles T., van der Heijden M.S., Castellano D., Galsky M.D., Loriot Y., Petrylak D.P., Ogawa O., Park S.H., Lee J.-L., De Giorgi U. (2020). Durvalumab alone and durvalumab plus tremelimumab versus chemotherapy in previously untreated patients with unresectable, locally advanced or metastatic urothelial carcinoma (DANUBE): A randomised, open-label, multicentre, phase 3 trial. Lancet Oncol..

[69] Garassino M.C., Cho B.-C., Kim J.-H., Mazières J., Vansteenkiste J., Lena H., Corral Jaime J., Gray J.E., Powderly J., Chouaid C. (2018). Durvalumab as third-line or later treatment for advanced non-small-cell lung cancer (ATLANTIC): An open-label, single-arm, phase 2 study. Lancet Oncol..

[70] Rizvi N.A., Cho B.C., Reinmuth N., Lee K.H., Luft A., Ahn M.-J., Van Den Heuvel M.M., Cobo M., Vicente D., Smolin A. (2020). Durvalumab with or Without Tremelimumab vs Standard Chemotherapy in First-line Treatment of Metastatic Non-Small Cell Lung Cancer: The MYSTIC Phase 3 Randomized Clinical Trial. JAMA Oncol..

[71] Theelen W.S.M.E., Peulen H.M.U., Lalezari F., Van Der Noort V., De Vries J.F., Aerts J.G.J.V., Dumoulin D.W., Bahce I., Niemeijer A.-L.N., De Langen A.J. (2019). Effect of Pembrolizumab after Stereotactic Body Radiotherapy vs Pembrolizumab Alone on Tumor Response in Patients with Advanced Non-Small Cell Lung Cancer: Results of the PEMBRO-RT Phase 2 Randomized Clinical Trial. JAMA Oncol..

[72] Powles T., Atkins M.B., Escudier B., Motzer R.J., Rini B.I., Fong L., Joseph R.W., Pal S.K., Sznol M., Hainsworth J. (2021). Efficacy and Safety of Atezolizumab Plus Bevacizumab Following Disease Progression on Atezolizumab or Sunitinib Monotherapy in Patients with Metastatic Renal Cell Carcinoma in IMmotion150: A Randomized Phase 2 Clinical Trial. Eur. Urol..

[73] Powles T., O’Donnell P.H., Massard C., Arkenau H.-T., Friedlander T.W., Hoimes C.J., Lee J.L., Ong M., Sridhar S.S., Vogelzang N.J. (2017). Efficacy and Safety of Durvalumab in Locally Advanced or Metastatic Urothelial Carcinoma: Updated Results From a Phase 1/2 Open-label Study. JAMA Oncol..

[74] Verschraegen C.F., Jerusalem G., McClay E.F., Iannotti N., Redfern C.H., Bennouna J., Chen F.L., Kelly K., Mehnert J., Morris J.C. (2020). Efficacy and safety of first-line avelumab in patients with advanced non-small cell lung cancer: Results from a phase Ib cohort of the JAVELIN Solid Tumor study. J. Immunother. Cancer.

[75] O’Day S.J., Maio M., Chiarion-Sileni V., Gajewski T.F., Pehamberger H., Bondarenko I.N., Queirolo P., Lundgren L., Mikhailov S., Roman L. (2010). Efficacy and safety of ipilimumab monotherapy in patients with pretreated advanced melanoma: A multicenter single-arm phase II study. Ann. Oncol. Off. J. Eur. Soc. Med. Oncol..

[76] Hida T., Nishio M., Nogami N., Ohe Y., Nokihara H., Sakai H., Satouchi M., Nakagawa K., Takenoyama M., Isobe H. (2017). Efficacy and safety of nivolumab in Japanese patients with advanced or recurrent squamous non-small cell lung cancer. Cancer Sci..

[77] Arrieta O., Barron F., Ramirez-Tirado L.A., Zatarain-Barron Z.L., Cardona A.F., Diaz-Garcia D., Yamamoto Ramos M., Mota-Vega B., Carmona A., Peralta Alvarez M.P. (2020). Efficacy and Safety of Pembrolizumab Plus Docetaxel vs Docetaxel Alone in Patients with Previously Treated Advanced Non-Small Cell Lung Cancer: The PROLUNG Phase 2 Randomized Clinical Trial. JAMA Oncol..

[78] Lebbé C., Meyer N., Mortier L., Marquez-Rodas I., Robert C., Rutkowski P., Menzies A.M., Eigentler T., Ascierto P.A., Smylie M. (2019). Evaluation of Two Dosing Regimens for Nivolumab in Combination With Ipilimumab in Patients With Advanced Melanoma: Results From the Phase IIIb/IV CheckMate 511 Trial. J. Clin. Oncol. Off. J. Am. Soc. Clin. Oncol..

[79] Namikawa K., Kiyohara Y., Takenouchi T., Uhara H., Uchi H., Yoshikawa S., Takatsuka S., Koga H., Wada N., Minami H. (2020). Final analysis of a phase II study of nivolumab in combination with ipilimumab for unresectable chemotherapy-naive advanced melanoma. J. Dermatol..

[80] Hamid O., Puzanov I., Dummer R., Schachter J., Daud A., Schadendorf D., Blank C., Cranmer L.D., Robert C., Pavlick A.C. (2017). Final analysis of a randomised trial comparing pembrolizumab versus investigator-choice chemotherapy for ipilimumab-refractory advanced melanoma. Eur. J. Cancer.

[81] de Azevedo S.J., de Melo A.C., Roberts L., Caro I., Xue C., Wainstein A. (2021). First-line atezolizumab monotherapy in patients with advanced BRAF(V600) wild-type melanoma. Pigment. Cell Melanoma Res..

[82] Paz-Ares L., Ciuleanu T.-E., Cobo M., Schenker M., Zurawski B., Menezes J., Richardet E., Bennouna J., Felip E., Juan-Vidal O. (2021). First-line nivolumab plus ipilimumab combined with two cycles of chemotherapy in patients with non-small-cell lung cancer (CheckMate 9LA): An international, randomised, open-label, phase 3 trial. Lancet Oncol..

[83] Balar A.V., Castellano D., O’Donnell P.H., Grivas P., Vuky J., Powles T., Plimack E.R., Hahn N.M., de Wit R., Pang L. (2017). First-line pembrolizumab in cisplatin-ineligible patients with locally advanced and unresectable or metastatic urothelial cancer (KEYNOTE-052): A multicentre, single-arm, phase 2 study. Lancet Oncol..

[84] Herbst R.S., Garon E.B., Kim D.-W., Cho B.C., Gervais R., Perez-Gracia J.L., Han J.-Y., Majem M., Forster M.D., Monnet I. (2021). Five Year Survival Update From KEYNOTE-010: Pembrolizumab Versus Docetaxel for Previously Treated, Programmed Death-Ligand 1-Positive Advanced NSCLC. J. Thorac. Oncol. Off. Publ. Int. Assoc. Study Lung Cancer.

[85] Robert C., Long G.V., Brady B., Dutriaux C., Di Giacomo A.M., Mortier L., Rutkowski P., Hassel J.C., McNeil C.M., Kalinka E.A. (2020). Five-year outcomes with nivolumab in patients with wild-type BRAF advanced melanoma. J. Clin. Oncol..

[86] Reck M., Rodriguez-Abreu D., Robinson A.G., Hui R., Csoszi T., Fulop A., Gottfried M., Peled N., Tafreshi A., Cuffe S. (2021). Five-Year Outcomes with Pembrolizumab Versus Chemotherapy for Metastatic Non-Small-Cell Lung Cancer with PD-L1 Tumor Proportion Score ++ 50%. J. Clin. Oncol..

[87] Hodi F.S., O’Day S.J., McDermott D.F., Weber R.W., Sosman J.A., Haanen J.B., Gonzalez R., Robert C., Schadendorf D., Hassel J.C. (2010). Improved survival with ipilimumab in patients with metastatic melanoma. N. Engl. J. Med..

[88] Ascierto P.A., Del Vecchio M., Robert C., Mackiewicz A., Chiarion-Sileni V., Arance A., Lebbe C., Bastholt L., Hamid O., Rutkowski P. (2017). Ipilimumab 10 mg/kg versus ipilimumab 3 mg/kg in patients with unresectable or metastatic melanoma: A randomised, double-blind, multicentre, phase 3 trial. Lancet Oncol..

[89] Di Giacomo A.M., Ascierto P.A., Pilla L., Santinami M., Ferrucci P.F., Giannarelli D., Marasco A., Rivoltini L., Simeone E., Nicoletti S.V. (2012). Ipilimumab and fotemustine in patients with advanced melanoma (NIBIT-M1): An open-label, single-arm phase 2 trial. Lancet Oncol..

[90] Aamdal E., Jacobsen K.D., Straume O., Kersten C., Herlofsen O., Karlsen J., Hussain I., Amundsen A., Dalhaug A., Nyakas M. (2022). Ipilimumab in a real-world population: A prospective Phase IV trial with long-term follow-up. Int. J. Cancer.

[91] Lynch T.J., Bondarenko I., Luft A., Serwatowski P., Barlesi F., Chacko R., Sebastian M., Neal J., Lu H., Cuillerot J.-M. (2012). Ipilimumab in combination with paclitaxel and carboplatin as first-line treatment in stage IIIB/IV non-small-cell lung cancer: Results from a randomized, double-blind, multicenter phase II study. J. Clin. Oncol..

[92] Margolin K., Ernstoff M.S., Hamid O., Lawrence D., McDermott D., Puzanov I., Wolchok J.D., Clark J.I., Sznol M., Logan T.F. (2012). Ipilimumab in patients with melanoma and brain metastases: An open-label, phase 2 trial. Lancet Oncol..

[93] Wolchok J.D., Neyns B., Linette G., Negrier S., Lutzky J., Thomas L., Waterfield W., Schadendorf D., Smylie M., Guthrie T. (2010). Ipilimumab monotherapy in patients with pretreated advanced melanoma: A randomised, double-blind, multicentre, phase 2, dose-ranging study. Lancet Oncol..

[94] Robert C., Thomas L., Bondarenko I., O’Day S., Weber J., Garbe C., Lebbe C., Baurain J.-F., Testori A., Grob J.-J. (2011). Ipilimumab plus dacarbazine for previously untreated metastatic melanoma. N. Engl. J. Med..

[95] Nishio M., Takahashi T., Yoshioka H., Nakagawa K., Fukuhara T., Yamada K., Ichiki M., Tanaka H., Seto T., Sakai H. (2019). KEYNOTE-025: Phase 1b study of pembrolizumab in Japanese patients with previously treated programmed death ligand 1-positive advanced non-small-cell lung cancer. Cancer Sci..

[96] Ma Y., Fang W., Zhang Y., Yang Y., Hong S., Zhao Y., Xie S., Ge J., Zhou H., Zhao H. (2020). KEYNOTE-032: A Randomized Phase I Study of Pembrolizumab in Chinese Patients with Advanced Non-Small Cell Lung Cancer. Oncologist.

[97] Lee C.-H., Shah A.Y., Rasco D., Rao A., Taylor M.H., Di Simone C., Hsieh J.J., Pinto A., Shaffer D.R., Girones Sarrio R. (2021). Lenvatinib plus pembrolizumab in patients with either treatment-naive or previously treated metastatic renal cell carcinoma (Study 111/KEYNOTE-146): A phase 1b/2 study. Lancet Oncol..

[98] Motzer R., Alekseev B., Rha S.-Y., Porta C., Eto M., Powles T., Grünwald V., Hutson T.E., Kopyltsov E., Méndez-Vidal M.J. (2021). Lenvatinib plus Pembrolizumab or Everolimus for Advanced Renal Cell Carcinoma. N. Engl. J. Med..

[99] Carlino M.S., Menzies A.M., Atkinson V., Cebon J.S., Jameson M.B., Fitzharris B.M., McNeil C.M., Hill A.G., Ribas A., Atkins M.B. (2020). Long-term Follow-up of Standard-Dose Pembrolizumab plus Reduced-Dose Ipilimumab in Patients with Advanced Melanoma: KEYNOTE-029 Part 1B. Clin. Cancer Res..

[100] Vuky J., Balar A.V., Castellano D., O’Donnell P.H., Grivas P., Bellmunt J., Powles T., Bajorin D., Hahn N.M., Savage M.J. (2020). Long-Term Outcomes in KEYNOTE-052: Phase II Study Investigating First-Line Pembrolizumab in Cisplatin-Ineligible Patients With Locally Advanced or Metastatic Urothelial Cancer. J. Clin. Oncol. Off. J. Am. Soc. Clin. Oncol..

[101] Tawbi H.A., Forsyth P.A., Hodi F.S., Algazi A.P., Hamid O., Lao C.D., Moschos S.J., Atkins M.B., Lewis K., Postow M.A. (2021). Long-term outcomes of patients with active melanoma brain metastases treated with combination nivolumab plus ipilimumab (CheckMate 204): Final results of an open-label, multicentre, phase 2 study. Lancet Oncol..

[102] Wolchok J.D., Chiarion-Sileni V., Gonzalez R., Grob J.-J., Rutkowski P., Lao C.D., Cowey C.L., Schadendorf D., Wagstaff J., Dummer R. (2022). Long-Term Outcomes With Nivolumab Plus Ipilimumab or Nivolumab Alone Versus Ipilimumab in Patients With Advanced Melanoma. J. Clin. Oncol. Off. J. Am. Soc. Clin. Oncol..

[103] Eggermont A.M.M., Blank C.U., Mandala M., Long G.V., Atkinson V.G., Dalle S., Haydon A.M., Meshcheryakov A., Khattak A., Carlino M.S. (2020). Longer Follow-Up Confirms Recurrence-Free Survival Benefit of Adjuvant Pembrolizumab in High-Risk Stage III Melanoma: Updated Results From the EORTC 1325-MG/KEYNOTE-054 Trial. J. Clin. Oncol. Off. J. Am. Soc. Clin. Oncol..

[104] Schwarze J.K., Garaud S., Jansen Y.J.L., Awada G., Vandersleyen V., Tijtgat J., de Wind A., Kristanto P., Seremet T., Willard-Gallo K. (2022). Low-Dose Nivolumab with or without Ipilimumab as Adjuvant Therapy Following the Resection of Melanoma Metastases: A Sequential Dual Cohort Phase II Clinical Trial. Cancers.

[105] Morgensztern D., Dols M.C., Ponce Aix S., Postmus P.E., Bennouna J., Fischer J.R., Juan-Vidal O., Stewart D.J., Ardizzoni A., Bhore R. (2020). nab-Paclitaxel Plus Durvalumab in Patients With Previously Treated Advanced Stage Non-small Cell Lung Cancer (ABOUND.2L+). Front. Oncol..

[106] Sharma P., Siefker-Radtke A., de Braud F., Basso U., Calvo E., Bono P., Morse M.A., Ascierto P.A., Lopez-Martin J., Brossart P. (2019). Nivolumab Alone and With Ipilimumab in Previously Treated Metastatic Urothelial Carcinoma: CheckMate 032 Nivolumab 1 mg/kg Plus Ipilimumab 3 mg/kg Expansion Cohort Results. J. Clin. Oncol. Off. J. Am. Soc. Clin. Oncol..

[107] Pelster M.S., Gruschkus S.K., Bassett R., Gombos D.S., Shephard M., Posada L., Glover M.S., Simien R., Diab A., Hwu P. (2021). Nivolumab and Ipilimumab in Metastatic Uveal Melanoma: Results From a Single-Arm Phase II Study. J. Clin. Oncol. Off. J. Am. Soc. Clin. Oncol..

[108] Postow M.A., Chesney J., Pavlick A.C., Robert C., Grossmann K., McDermott D., Linette G.P., Meyer N., Giguere J.K., Agarwala S.S. (2015). Nivolumab and ipilimumab versus ipilimumab in untreated melanoma. N. Engl. J. Med..

[109] Motzer R.J., Rini B.I., McDermott D.F., Redman B.G., Kuzel T.M., Harrison M.R., Vaishampayan U.N., Drabkin H.A., George S., Logan T.F. (2015). Nivolumab for metastatic renal cell carcinoma: Results of a randomized phase II trial. J. Clin. Oncol..

[110] Lee J.S., Lee K.H., Cho E.K., Kim D.-W., Kim S.-W., Kim J.-H., Cho B.C., Kang J.H., Han J.-Y., Min Y.J. (2018). Nivolumab in advanced non-small-cell lung cancer patients who failed prior platinum-based chemotherapy. Lung Cancer Amst. Neth..

[111] Galsky M.D., Saci A., Szabo P.M., Han G.C., Grossfeld G., Collette S., Siefker-Radtke A., Necchi A., Sharma P. (2020). Nivolumab in Patients with Advanced Platinum-resistant Urothelial Carcinoma: Efficacy, Safety, and Biomarker Analyses with Extended Follow-up from CheckMate 275. Clin. Cancer Res. Off. J. Am. Assoc. Cancer Res..

[112] Gettinger S., Rizvi N.A., Chow L.Q., Borghaei H., Brahmer J., Ready N., Gerber D.E., Shepherd F.A., Antonia S., Goldman J.W. (2016). Nivolumab Monotherapy for First-Line Treatment of Advanced Non-Small-Cell Lung Cancer. J. Clin. Oncol. Off. J. Am. Soc. Clin. Oncol..

[113] Choueiri T.K., Powles T., Burotto M., Escudier B., Bourlon M.T., Zurawski B., Juarez V.M.O., Hsieh J.J., Basso U., Shah A.Y. (2021). Nivolumab plus Cabozantinib versus Sunitinib for Advanced Renal-Cell Carcinoma. N. Engl. J. Med..

[114] Hellmann M.D., Rizvi N.A., Goldman J.W., Gettinger S.N., Borghaei H., Brahmer J.R., Ready N.E., Gerber D.E., Chow L.Q., Juergens R.A. (2017). Nivolumab plus ipilimumab as first-line treatment for advanced non-small-cell lung cancer (CheckMate 012): Results of an open-label, phase 1, multicohort study. Lancet Oncol..

[115] Piulats J.M., Espinosa E., de la Cruz Merino L., Varela M., Alonso Carrión L., Martín-Algarra S., López Castro R., Curiel T., Rodríguez-Abreu D., Redrado M. (2021). Nivolumab Plus Ipilimumab for Treatment-Naïve Metastatic Uveal Melanoma: An Open-Label, Multicenter, Phase II Trial by the Spanish Multidisciplinary Melanoma Group (GEM-1402). J. Clin. Oncol. Off. J. Am. Soc. Clin. Oncol..

[116] Hellmann M.D., Paz-Ares L., Bernabe Caro R., Zurawski B., Kim S.-W., Carcereny Costa E., Park K., Alexandru A., Lupinacci L., de la Mora Jimenez E. (2019). Nivolumab plus Ipilimumab in Advanced Non-Small-Cell Lung Cancer. N. Engl. J. Med..

[117] Callahan M.K., Kluger H., Postow M.A., Segal N.H., Lesokhin A., Atkins M.B., Kirkwood J.M., Krishnan S., Bhore R., Horak C. (2018). Nivolumab Plus Ipilimumab in Patients With Advanced Melanoma: Updated Survival, Response, and Safety Data in a Phase I Dose-Escalation Study. J. Clin. Oncol. Off. J. Am. Soc. Clin. Oncol..

[118] Albiges L., Tannir N.M., Burotto M., McDermott D., Plimack E.R., Barthélémy P., Porta C., Powles T., Donskov F., George S. (2020). Nivolumab plus ipilimumab versus sunitinib for first-line treatment of advanced renal cell carcinoma: Extended 4-year follow-up of the phase III CheckMate 214 trial. ESMO Open.

[119] Gettinger S.N., Redman M.W., Bazhenova L., Hirsch F.R., Mack P.C., Schwartz L.H., Bradley J.D., Stinchcombe T.E., Leighl N.B., Ramalingam S.S. (2021). Nivolumab plus Ipilimumab vs Nivolumab for Previously Treated Patients with Stage IV Squamous Cell Lung Cancer: The Lung-MAP S1400I Phase 3 Randomized Clinical Trial. JAMA Oncol..

[120] Chen Y.-M., Chih-Hsin Yang J., Su W.-C., Chong I.-W., Hsia T.-C., Lin M.-C., Chang G.-C., Chiu C.-H., Ho C.-C., Wu S.-Y. (2020). Nivolumab safety and efficacy in advanced, platinum-resistant, non-small cell lung cancer, radical radiotherapy-ineligible patients: A phase II study in Taiwan. J. Formos. Med. Assoc..

[121] Lu S., Wang J., Cheng Y., Mok T., Chang J., Zhang L., Feng J., Tu H.-Y., Wu L., Zhang Y. (2021). Nivolumab versus docetaxel in a predominantly Chinese patient population with previously treated advanced non-small cell lung cancer: 2-year follow-up from a randomized, open-label, phase 3 study (CheckMate 078). Lung Cancer.

[122] Wu Y.-L., Lu S., Cheng Y., Zhou C., Wang J., Mok T., Zhang L., Tu H.-Y., Wu L., Feng J. (2019). Nivolumab Versus Docetaxel in a Predominantly Chinese Patient Population With Previously Treated Advanced NSCLC: CheckMate 078 Randomized Phase III Clinical Trial. J. Thorac. Oncol..

[123] Borghaei H., Paz-Ares L., Horn L., Spigel D.R., Steins M., Ready N.E., Chow L.Q., Vokes E.E., Felip E., Holgado E. (2015). Nivolumab versus docetaxel in advanced nonsquamous non-small-cell lung cancer. N. Engl. J. Med..

[124] Brahmer J., Reckamp K.L., Baas P., Crino L., Eberhardt W.E.E., Poddubskaya E., Antonia S., Pluzanski A., Vokes E.E., Holgado E. (2015). Nivolumab versus docetaxel in advanced squamous-cell non-small-cell lung cancer. N. Engl. J. Med..

[125] Motzer R.J., Escudier B., George S., Hammers H.J., Srinivas S., Tykodi S.S., Sosman J.A., Plimack E.R., Procopio G., McDermott D.F. (2020). Nivolumab versus everolimus in patients with advanced renal cell carcinoma: Updated results with long-term follow-up of the randomized, open-label, phase 3 CheckMate 025 trial. Cancer.

[126] Sugawara S., Lee J.-S., Kang J.-H., Kim H.R., Inui N., Hida T., Lee K.H., Yoshida T., Tanaka H., Yang C.-T. (2021). Nivolumab with carboplatin, paclitaxel, and bevacizumab for first-line treatment of advanced nonsquamous non-small-cell lung cancer. Ann. Oncol..

[127] Zimmer L., Eigentler T.K., Kiecker F., Simon J., Utikal J., Mohr P., Berking C., Kampgen E., Dippel E., Stadler R. (2015). Open-label, multicenter, single-arm phase II DeCOG-study of ipilimumab in pretreated patients with different subtypes of metastatic melanoma. J. Transl. Med..

[128] McDermott D.F., Lee J.-L., Bjarnason G.A., Larkin J.M.G., Gafanov R.A., Kochenderfer M.D., Jensen N.V., Donskov F., Malik J., Poprach A. (2021). Open-Label, Single-Arm Phase II Study of Pembrolizumab Monotherapy as First-Line Therapy in Patients With Advanced Clear Cell Renal Cell Carcinoma. J. Clin. Oncol. Off. J. Am. Soc. Clin. Oncol..

[129] McDermott D.F., Lee J.-L., Ziobro M., Suarez C., Langiewicz P., Matveev V.B., Wiechno P., Gafanov R.A., Tomczak P., Pouliot F. (2021). Open-Label, Single-Arm, Phase II Study of Pembrolizumab Monotherapy as First-Line Therapy in Patients With Advanced Non-Clear Cell Renal Cell Carcinoma. J. Clin. Oncol. Off. J. Am. Soc. Clin. Oncol..

[130] McKay R.R., McGregor B.A., Xie W., Braun D.A., Wei X., Kyriakopoulos C.E., Zakharia Y., Maughan B.L., Rose T.L., Stadler W.M. (2020). Optimized Management of Nivolumab and Ipilimumab in Advanced Renal Cell Carcinoma: A Response-Based Phase II Study (OMNIVORE). J. Clin. Oncol. Off. J. Am. Soc. Clin. Oncol..

[131] Gettinger S.N., Horn L., Gandhi L., Spigel D.R., Antonia S.J., Rizvi N.A., Powderly J.D., Heist R.S., Carvajal R.D., Jackman D.M. (2015). Overall survival and long-term safety of nivolumab (anti-programmed death 1 antibody, BMS-936558, ONO-4538) in patients with previously treated advanced non-small-cell lung cancer. J. Clin. Oncol..

[132] Larkin J., Minor D., D’Angelo S., Neyns B., Smylie M., Miller W.H.J., Gutzmer R., Linette G., Chmielowski B., Lao C.D. (2018). Overall Survival in Patients With Advanced Melanoma Who Received Nivolumab Versus Investigator’s Choice Chemotherapy in CheckMate 037: A Randomized, Controlled, Open-Label Phase III Trial. J. Clin. Oncol. Off. J. Am. Soc. Clin. Oncol..

[133] Antonia S.J., Villegas A., Daniel D., Vicente D., Murakami S., Hui R., Kurata T., Chiappori A., Lee K.H., De Wit M. (2018). Overall survival with durvalumab after chemoradiotherapy in stage III NSCLC. N. Engl. J. Med..

[134] Powles T., Csőszi T., Özgüroğlu M., Matsubara N., Géczi L., Cheng S.Y.-S., Fradet Y., Oudard S., Vulsteke C., Morales Barrera R. (2021). Pembrolizumab alone or combined with chemotherapy versus chemotherapy as first-line therapy for advanced urothelial carcinoma (KEYNOTE-361): A randomised, open-label, phase 3 trial. Lancet Oncol..

[135] Giannatempo P., Raggi D., Marandino L., Bandini M., Farè E., Calareso G., Colecchia M., Gallina A., Ross J.S., Alessi A. (2020). Pembrolizumab and nab-paclitaxel as salvage therapy for platinum-treated, locally advanced or metastatic urothelial carcinoma: Interim results of the open-label, single-arm, phase II PEANUT study. Ann. Oncol. Off. J. Eur. Soc. Med. Oncol..

[136] Bellmunt J., de Wit R., Vaughn D.J., Fradet Y., Lee J.-L., Fong L., Vogelzang N.J., Climent M.A., Petrylak D.P., Choueiri T.K. (2017). Pembrolizumab as Second-Line Therapy for Advanced Urothelial Carcinoma. N. Engl. J. Med..

[137] Goldberg S.B., Schalper K.A., Gettinger S.N., Mahajan A., Herbst R.S., Chiang A.C., Lilenbaum R., Wilson F.H., Omay S.B., Yu J.B. (2020). Pembrolizumab for management of patients with NSCLC and brain metastases: Long-term results and biomarker analysis from a non-randomised, open-label, phase 2 trial. Lancet Oncol..

[138] Garon E.B., Rizvi N.A., Hui R., Leighl N., Balmanoukian A.S., Eder J.P., Patnaik A., Aggarwal C., Gubens M., Horn L. (2015). Pembrolizumab for the treatment of non-small-cell lung cancer. N. Engl. J. Med..

[139] Gubens M.A., Sequist L.V., Stevenson J.P., Powell S.F., Villaruz L.C., Gadgeel S.M., Langer C.J., Patnaik A., Borghaei H., Jalal S.I. (2019). Pembrolizumab in combination with ipilimumab as second-line or later therapy for advanced non-small-cell lung cancer: KEYNOTE-021 cohorts D and H. Lung Cancer.

[140] Middleton G., Brock K., Savage J., Mant R., Summers Y., Connibear J., Shah R., Ottensmeier C., Shaw P., Lee S.-M. (2020). Pembrolizumab in patients with non-small-cell lung cancer of performance status 2 (PePS2): A single arm, phase 2 trial. Lancet Respir. Med..

[141] Balar A.V., Kamat A.M., Kulkarni G.S., Uchio E.M., Boormans J.L., Roumiguié M., Krieger L.E.M., Singer E.A., Bajorin D.F., Grivas P. (2021). Pembrolizumab monotherapy for the treatment of high-risk non-muscle-invasive bladder cancer unresponsive to BCG (KEYNOTE-057): An open-label, single-arm, multicentre, phase 2 study. Lancet Oncol..

[142] Rini B.I., Plimack E.R., Stus V., Gafanov R., Hawkins R., Nosov D., Pouliot F., Alekseev B., Soulières D., Melichar B. (2019). Pembrolizumab plus Axitinib versus Sunitinib for Advanced Renal-Cell Carcinoma. N. Engl. J. Med..

[143] Paz-Ares L., Luft A., Vicente D., Tafreshi A., Gümüş M., Mazières J., Hermes B., Çay Şenler F., Csőszi T., Fülöp A. (2018). Pembrolizumab plus Chemotherapy for Squamous Non-Small-Cell Lung Cancer. N. Engl. J. Med..

[144] Olson D.J., Eroglu Z., Brockstein B., Poklepovic A.S., Bajaj M., Babu S., Hallmeyer S., Velasco M., Lutzky J., Higgs E. (2021). Pembrolizumab Plus Ipilimumab Following Anti-PD-1/L1 Failure in Melanoma. J. Clin. Oncol. Off. J. Am. Soc. Clin. Oncol..

[145] Reck M., Rodríguez-Abreu D., Robinson A.G., Hui R., Csőszi T., Fülöp A., Gottfried M., Peled N., Tafreshi A., Cuffe S. (2016). Pembrolizumab versus Chemotherapy for PD-L1-Positive Non-Small-Cell Lung Cancer. N. Engl. J. Med..

[146] Mok T.S.K., Wu Y.-L., Kudaba I., Kowalski D.M., Cho B.C., Turna H.Z., Castro G., Srimuninnimit V., Laktionov K.K., Bondarenko I. (2019). Pembrolizumab versus chemotherapy for previously untreated, PD-L1-expressing, locally advanced or metastatic non-small-cell lung cancer (KEYNOTE-042): A randomised, open-label, controlled, phase 3 trial. Lancet.

[147] Robert C., Ribas A., Schachter J., Arance A., Grob J.-J., Mortier L., Daud A., Carlino M.S., McNeil C.M., Lotem M. (2019). Pembrolizumab versus ipilimumab in advanced melanoma (KEYNOTE-006): Post-hoc 5-year results from an open-label, multicentre, randomised, controlled, phase 3 study. Lancet Oncol..

[148] Rodríguez-Abreu D., Powell S.F., Hochmair M.J., Gadgeel S., Esteban E., Felip E., Speranza G., De Angelis F., Dómine M., Cheng S.Y. (2021). Pemetrexed plus platinum with or without pembrolizumab in patients with previously untreated metastatic nonsquamous NSCLC: Protocol-specified final analysis from KEYNOTE-189. Ann. Oncol. Off. J. Eur. Soc. Med. Oncol..

[149] Jamal R., Lapointe R., Cocolakis E., Thébault P., Kazemi S., Friedmann J.E., Dionne J., Cailhier J.-F., Bélanger K., Ayoub J.-P. (2017). Peripheral and local predictive immune signatures identified in a phase II trial of ipilimumab with carboplatin/paclitaxel in unresectable stage III or stage IV melanoma. J. Immunother. Cancer.

[150] Yamazaki N., Takenouchi T., Fujimoto M., Ihn H., Uchi H., Inozume T., Kiyohara Y., Uhara H., Nakagawa K., Furukawa H. (2017). Phase 1b study of pembrolizumab (MK-3475; anti-PD-1 monoclonal antibody) in Japanese patients with advanced melanoma (KEYNOTE-041). Cancer Chemother. Pharmacol..

[151] Galsky M.D., Wang H., Hahn N.M., Twardowski P., Pal S.K., Albany C., Fleming M.T., Starodub A., Hauke R.J., Yu M. (2018). Phase 2 Trial of Gemcitabine, Cisplatin, plus Ipilimumab in Patients with Metastatic Urothelial Cancer and Impact of DNA Damage Response Gene Mutations on Outcomes. Eur. Urol..

[152] Weber J., Gibney G., Kudchadkar R., Yu B., Cheng P., Martinez A.J., Kroeger J., Richards A., McCormick L., Moberg V. (2016). Phase I/II Study of Metastatic Melanoma Patients Treated with Nivolumab Who Had Progressed after Ipilimumab. Cancer Immunol. Res..

[153] Dudek A.Z., Liu L.C., Gupta S., Logan T.F., Singer E.A., Joshi M., Zakharia Y.N., Lang J.M., Schwarz J.K., Al-Janadi A. (2020). Phase Ib/II clinical trial of pembrolizumab with bevacizumab for metastatic renal cell carcinoma: BTCRC-GU14-003. J. Clin. Oncol..

[154] Zimmer L., Vaubel J., Mohr P., Hauschild A., Utikal J., Simon J., Garbe C., Herbst R., Enk A., Kämpgen E. (2015). Phase II DeCOG-study of ipilimumab in pretreated and treatment-naïve patients with metastatic uveal melanoma. PLoS ONE.

[155] Seto T., Nosaki K., Shimokawa M., Toyozawa R., Sugawara S., Hayashi H., Murakami H., Kato T., Niho S., Saka H. (2022). Phase II study of atezolizumab with bevacizumab for non-squamous non-small cell lung cancer with high PD-L1 expression (@Be Study). J. Immunother. Cancer.

[156] Peters S., Gettinger S., Johnson M.L., Jänne P.A., Garassino M.C., Christoph D., Toh C.K., Rizvi N.A., Chaft J.E., Carcereny Costa E. (2017). Phase II Trial of Atezolizumab As First-Line or Subsequent Therapy for Patients With Programmed Death-Ligand 1-Selected Advanced Non-Small-Cell Lung Cancer (BIRCH). J. Clin. Oncol. Off. J. Am. Soc. Clin. Oncol..

[157] Ribas A., Kefford R., Marshall M.A., Punt C.J.A., Haanen J.B., Marmol M., Garbe C., Gogas H., Schachter J., Linette G. (2013). Phase III randomized clinical trial comparing tremelimumab with standard-of-care chemotherapy in patients with advanced melanoma. J. Clin. Oncol..

[158] Tarhini A.A., Lee S.J., Hodi F.S., Rao U.N.M., Cohen G.I., Hamid O., Hutchins L.F., Sosman J.A., Kluger H.M., Eroglu Z. (2020). Phase III Study of Adjuvant Ipilimumab (3 or 10 mg/kg) Versus High-Dose Interferon Alfa-2b for Resected High-Risk Melanoma: North American Intergroup E1609. J. Clin. Oncol. Off. J. Am. Soc. Clin. Oncol..

[159] Govindan R., Szczesna A., Ahn M.-J., Schneider C.-P., Mella P.F.G., Barlesi F., Han B., Ganea D.E., Pawel J.V., Vladimirov V. (2017). Phase III trial of ipilimumab combined with paclitaxel and carboplatin in advanced squamous non-small-cell lung cancer. J. Clin. Oncol..

[160] Choueiri T.K., Larkin J., Oya M., Thistlethwaite F., Martignoni M., Nathan P., Powles T., McDermott D., Robbins P.B., Chism D.D. (2018). Preliminary results for avelumab plus axitinib as first-line therapy in patients with advanced clear-cell renal-cell carcinoma (JAVELIN Renal 100): An open-label, dose-finding and dose-expansion, phase 1b trial. Lancet Oncol..

[161] Sternberg C.N., Loriot Y., James N., Choy E., Castellano D., Lopez-Rios F., Banna G.L., De Giorgi U., Masini C., Bamias A. (2019). Primary Results from SAUL, a Multinational Single-arm Safety Study of Atezolizumab Therapy for Locally Advanced or Metastatic Urothelial or Nonurothelial Carcinoma of the Urinary Tract. Eur. Urol..

[162] Ardizzoni A., Azevedo S., Rubio-Viqueira B., Rodríguez-Abreu D., Alatorre-Alexander J., Smit H.J.M., Yu J., Syrigos K., Trunzer K., Patel H. (2021). Primary results from TAIL: A global single-arm safety study of atezolizumab monotherapy in a diverse population of patients with previously treated advanced non-small cell lung cancer. J. Immunother. Cancer.

[163] Eggermont A.M.M., Chiarion-sileni V., Grob J.-J., Dummer R., Wolchok J.D., Schmidt H., Hamid O., Robert C., Ascierto P.A., Richards J.M. (2016). Prolonged survival in stage III melanoma with ipilimumab adjuvant therapy. N. Engl. J. Med..

[164] Chesney J., Puzanov I., Collichio F., Singh P., Milhem M.M., Glaspy J., Hamid O., Ross M., Friedlander P., Garbe C. (2018). Randomized, open-label phase II study evaluating the efficacy and safety of talimogene laherparepvec in combination with ipilimumab versus ipilimumab alone in patients with advanced, unresectable melanoma. J. Clin. Oncol..

[165] Tawbi H.A., Schadendorf D., Lipson E.J., Ascierto P.A., Matamala L., Castillo Gutiérrez E., Rutkowski P., Gogas H.J., Lao C.D., De Menezes J.J. (2022). Relatlimab and Nivolumab versus Nivolumab in Untreated Advanced Melanoma. N. Engl. J. Med..

[166] McGregor B.A., McKay R.R., Braun D.A., Werner L., Gray K., Flaifel A., Signoretti S., Hirsch M.S., Steinharter J.A., Bakouny Z. (2020). Results of a Multicenter Phase II Study of Atezolizumab and Bevacizumab for Patients With Metastatic Renal Cell Carcinoma With Variant Histology and/or Sarcomatoid Features. J. Clin. Oncol. Off. J. Am. Soc. Clin. Oncol..

[167] Horn L., Gettinger S.N., Gordon M.S., Herbst R.S., Gandhi L., Felip E., Sequist L.V., Spigel D.R., Antonia S.J., Balmanoukian A. (2018). Safety and clinical activity of atezolizumab monotherapy in metastatic non-small-cell lung cancer: Final results from a phase I study. Eur. J. Cancer Oxf. Engl. 1990.

[168] Flippot R., Dalban C., Laguerre B., Borchiellini D., Gravis G., Négrier S., Chevreau C., Joly F., Geoffrois L., Ladoire S. (2019). Safety and Efficacy of Nivolumab in Brain Metastases From Renal Cell Carcinoma: Results of the GETUG-AFU 26 NIVOREN Multicenter Phase II Study. J. Clin. Oncol. Off. J. Am. Soc. Clin. Oncol..

[169] Hammers H.J., Plimack E.R., Infante J.R., Rini B.I., McDermott D.F., Lewis L.D., Voss M.H., Sharma P., Pal S.K., Razak A.R.A. (2017). Safety and Efficacy of Nivolumab in Combination With Ipilimumab in Metastatic Renal Cell Carcinoma: The CheckMate 016 Study. J. Clin. Oncol. Off. J. Am. Soc. Clin. Oncol..

[170] Amin A., Plimack E.R., Ernstoff M.S., Lewis L.D., Bauer T.M., McDermott D.F., Carducci M., Kollmannsberger C., Rini B.I., Heng D.Y.C. (2018). Safety and efficacy of nivolumab in combination with sunitinib or pazopanib in advanced or metastatic renal cell carcinoma: The CheckMate 016 study. J. Immunother. Cancer.

[171] McFarlane J.J., Kochenderfer M.D., Olsen M.R., Bauer T.M., Molina A., Hauke R.J., Reeves J.A., Babu S., Van Veldhuizen P., Somer B. (2020). Safety and Efficacy of Nivolumab in Patients With Advanced Clear Cell Renal Cell Carcinoma: Results From the Phase IIIb/IV CheckMate 374 Study. Clin. Genitourin. Cancer.

[172] Vogelzang N.J., Olsen M.R., McFarlane J.J., Arrowsmith E., Bauer T.M., Jain R.K., Somer B., Lam E.T., Kochenderfer M.D., Molina A. (2020). Safety and Efficacy of Nivolumab in Patients With Advanced Non-Clear Cell Renal Cell Carcinoma: Results From the Phase IIIb/IV CheckMate 374 Study. Clin. Genitourin. Cancer.

[173] Nathan P., Ascierto P.A., Haanen J., Espinosa E., Demidov L., Garbe C., Guida M., Lorigan P., Chiarion-Sileni V., Gogas H. (2019). Safety and efficacy of nivolumab in patients with rare melanoma subtypes who progressed on or after ipilimumab treatment: A single-arm, open-label, phase II study (CheckMate 172). Eur. J. Cancer Oxf. Engl. 1990.

[174] Tykodi S.S., Gordan L.N., Alter R.S., Arrowsmith E., Harrison M.R., Percent I., Singal R., Van Veldhuizen P., George D.J., Hutson T. (2022). Safety and efficacy of nivolumab plus ipilimumab in patients with advanced non-clear cell renal cell carcinoma: Results from the phase 3b/4 CheckMate 920 trial. J. Immunother. Cancer.

[175] Perets R., Bar J., Rasco D.W., Ahn M.-J., Yoh K., Kim D.-W., Nagrial A., Satouchi M., Lee D.H., Spigel D.R. (2021). Safety and efficacy of quavonlimab, a novel anti-CTLA-4 antibody (MK-1308), in combination with pembrolizumab in first-line advanced non-small-cell lung cancer. Ann. Oncol. Off. J. Eur. Soc. Med. Oncol..

[176] Ribas A., Chesney J.A., Gordon M.S., Abernethy A.P., Logan T.F., Lawson D.H., Chmielowksi B., Glaspy J.A., Lewis K., Huang B. (2012). Safety profile and pharmacokinetic analyses of the anti-CTLA4 antibody tremelimumab administered as a one hour infusion. J. Transl. Med..

[177] Waterhouse D., Horn L., Reynolds C., Spigel D., Chandler J., Mekhail T., Mohamed M., Creelan B., Blankstein K.B., Nikolinakos P. (2018). Safety profile of nivolumab administered as 30-min infusion: Analysis of data from CheckMate 153. Cancer Chemother. Pharmacol..

[178] Hamid O., Molinero L., Bolen C.R., Sosman J.A., Muñoz-Couselo E., Kluger H.M., McDermott D.F., Powderly J.D., Sarkar I., Ballinger M. (2019). Safety, Clinical Activity, and Biological Correlates of Response in Patients with Metastatic Melanoma: Results from a Phase I Trial of Atezolizumab. Clin. Cancer Res. Off. J. Am. Assoc. Cancer Res..

[179] Weber J.S., Kudchadkar R.R., Yu B., Gallenstein D., Horak C.E., Inzunza H.D., Zhao X., Martinez A.J., Wang W., Gibney G. (2013). Safety, efficacy, and biomarkers of nivolumab with vaccine in ipilimumab-refractory or -naive melanoma. J. Clin. Oncol..

[180] Spigel D.R., McCleod M., Jotte R.M., Einhorn L., Horn L., Waterhouse D.M., Creelan B., Babu S., Leighl N.B., Chandler J.C. (2019). Safety, Efficacy, and Patient-Reported Health-Related Quality of Life and Symptom Burden with Nivolumab in Patients with Advanced Non-Small Cell Lung Cancer, Including Patients Aged 70 Years or Older or with Poor Performance Status (CheckMate 153). J. Thorac. Oncol..

[181] Weber J.S., Gibney G., Sullivan R.J., Sosman J.A., Slingluff C.L., Lawrence D.P., Logan T.F., Schuchter L.M., Nair S., Fecher L. (2016). Sequential administration of nivolumab and ipilimumab with a planned switch in patients with advanced melanoma (CheckMate 064): An open-label, randomised, phase 2 trial. Lancet Oncol..

[182] Long G.V., Atkinson V., Cebon J.S., Jameson M.B., Fitzharris B.M., McNeil C.M., Hill A.G., Ribas A., Atkins M.B., Thompson J.A. (2017). Standard-dose pembrolizumab in combination with reduced-dose ipilimumab for patients with advanced melanoma (KEYNOTE-029): An open-label, phase 1b trial. Lancet Oncol..

[183] Long G.V., Robert C., Butler M.O., Couture F., Carlino M.S., O’Day S., Atkinson V., Cebon J.S., Brown M.P., Dalle S. (2021). Standard-dose pembrolizumab plus alternate-dose ipilimumab in advanced melanoma: KEYNOTE-029 cohort 1C, a phase 2 randomized study of two dosing schedules. Clin. Cancer Res..

[184] McDermott D.F., Choueiri T.K., Puzanov I., Hodi S., Drake C.G., Brahmer J.R., Hammers H.J., Topalian S.L., Pardoll D.M., Sznol M. (2015). Survival, durable response, and long-term safety in patients with previously treated advanced renal cell carcinoma receiving nivolumab. J. Clin. Oncol..

[185] Borghaei H., Redman M.W., Kelly K., Waqar S.N., Robert F., Kiefer G.J., Stella P.J., Minichiello K., Gandara D.R., Herbst R.S. (2021). SWOG S1400A (NCT02154490): A Phase II Study of Durvalumab for Patients With Previously Treated Stage IV or Recurrent Squamous Cell Lung Cancer (Lung-MAP Sub-study). Clin. Lung Cancer.

[186] Wang J., Lu S., Yu X., Hu Y., Sun Y., Wang Z., Zhao J., Yu Y., Hu C., Yang K. (2021). Tislelizumab plus chemotherapy vs chemotherapy alone as first-line treatment for advanced squamous non-small-cell lung cancer a phase 3 randomized clinical trial. JAMA Oncol..

[187] Reck M., Rodriguez-Abreu D., Robinson A.G., Hui R., Csoszi T., Fulop A., Gottfried M., Peled N., Tafreshi A., Cuffe S. (2019). Updated analysis of KEYNOTE-024: Pembrolizumab versus platinum-based chemotherapy for advanced non-small-cell lung cancer with PD-L1 tumor proportion score of 50% or greater. J. Clin. Oncol..

[188] Fehrenbacher L., von Pawel J., Park K., Rittmeyer A., Gandara D.R., Ponce Aix S., Han J.-Y., Gadgeel S.M., Hida T., Cortinovis D.L. (2018). Updated Efficacy Analysis Including Secondary Population Results for OAK: A Randomized Phase III Study of Atezolizumab versus Docetaxel in Patients with Previously Treated Advanced Non-Small Cell Lung Cancer. J. Thorac. Oncol..

[189] Choueiri T.K., Motzer R.J., Rini B.I., Haanen J., Campbell M.T., Venugopal B., Kollmannsberger C., Gravis-Mescam G., Uemura M., Lee J.L. (2020). Updated efficacy results from the JAVELIN Renal 101 trial: First-line avelumab plus axitinib versus sunitinib in patients with advanced renal cell carcinoma. Ann. Oncol..

[190] Jassem J., de Marinis F., Giaccone G., Vergnenegre A., Barrios C.H., Morise M., Felip E., Oprean C., Kim Y.-C., Andric Z. (2021). Updated Overall Survival Analysis From IMpower110: Atezolizumab Versus Platinum-Based Chemotherapy in Treatment-Naive Programmed Death-Ligand 1-Selected NSCLC. J. Thorac. Oncol..

[191] Owen C.N., Bai X., Quah T., Lo S.N., Allayous C., Callaghan S., Martínez-Vila C., Wallace R., Bhave P., Reijers I.L.M. (2021). Delayed immune-related adverse events with anti-PD-1-based immunotherapy in melanoma. Ann. Oncol. Off. J. Eur. Soc. Med. Oncol..

[192] Patrinely J.R., Johnson R., Lawless A.R., Bhave P., Sawyers A., Dimitrova M., Yeoh H.L., Palmeri M., Ye F., Fan R. (2021). Chronic Immune-Related Adverse Events Following Adjuvant Anti-PD-1 Therapy for High-risk Resected Melanoma. JAMA Oncol..

