# Supplementary material for: Incidence of Cutaneous Immune-Related Adverse Events and Outcomes in Immune Checkpoint Inhibitor-Containing Regimens: A Systematic Review and Meta-Analysis
Source: Cancers (Basel). 2024 Jan 13;16(2):340. doi: 10.3390/cancers16020340 (PMC10814132; doi:10.3390/cancers16020340)
Supplement: Supplementary file 1 [file cancers-16-00340-s001.zip › cancers-2731846-supplementary.pdf]

Study	Events	Total	Incidence	Random, 95% CI
Arrieta 2020	0	40	0.000	[0.000; 0.088]
Barlesi 2018	5	393	0.013	[0.004; 0.029]
Waterhouse 2018	6	368	0.016	[0.006; 0.035]
Garassino 2018	2	111	0.018	[0.002; 0.064]
Brahmer 2015	3	131	0.023	[0.005; 0.065]
Wolchok 2010	2	72	0.028	[0.003; 0.097]
Apolo 2020	8	249	0.032	[0.014; 0.062]
Waterhouse 2018	14	369	0.038	[0.021; 0.063]
Spigel 2019	64	1426	0.045	[0.035; 0.057]
Felip 2020	43	811	0.053	[0.039; 0.071]
Antonia 2019	16	304	0.053	[0.030; 0.084]
Verschraegen 2020	8	156	0.051	[0.022; 0.099]
Powles 2017	10	191	0.052	[0.025; 0.094]
Middleton 2020	3	60	0.050	[0.010; 0.139]
Fillipot 2019	2	39	0.051	[0.006; 0.173]
Rizvi 2015	7	117	0.060	[0.024; 0.119]
Ardizzoni 2021	41	615	0.067	[0.048; 0.089]
Garassino 2018	18	265	0.068	[0.041; 0.105]
Fillipot 2019	2	34	0.059	[0.007; 0.197]
Planchard 2020	8	117	0.068	[0.030; 0.130]
Mok 2019	46	636	0.072	[0.053; 0.095]
Pal 2021	2	32	0.062	[0.008; 0.208]
Nathan 2019	78	1008	0.077	[0.062; 0.096]
Wu 2019	28	337	0.083	[0.056; 0.118]
Borghaei 2015	24	287	0.084	[0.054; 0.122]
Galsky 2020	30	354	0.085	[0.058; 0.119]
Felip 2021	43	495	0.087	[0.064; 0.115]
Rizvi 2020	32	369	0.087	[0.060; 0.120]
Gettinger 2015	11	129	0.085	[0.043; 0.147]
Giannatempo 2020	6	70	0.086	[0.032; 0.177]
Sternberg 2019	91	997	0.091	[0.074; 0.111]
Peters 2017	65	659	0.099	[0.077; 0.124]
Motzer 2015	5	54	0.093	[0.031; 0.203]
Rosenberg 2016	31	310	0.100	[0.069; 0.139]
Zimmer 2015	5	53	0.094	[0.031; 0.207]
deAzevedo 2021	5	52	0.096	[0.032; 0.210]
Ma 2020	4	42	0.095	[0.027; 0.226]
Powles 2020	36	345	0.104	[0.074; 0.142]
Herbst 2021	72	682	0.106	[0.084; 0.131]
Garon 2015	53	495	0.107	[0.081; 0.138]
Motzer 2015	6	59	0.102	[0.038; 0.208]
Zimmer 2015	11	103	0.107	[0.055; 0.183]
Balar 2017	13	119	0.109	[0.059; 0.180]
Balar 2021	11	101	0.109	[0.056; 0.187]
West 2019	53	473	0.112	[0.085; 0.144]
Zimmer 2020	6	56	0.107	[0.040; 0.219]
Amdal 2022	17	151	0.113	[0.067; 0.174]
Planchard 2020	20	173	0.116	[0.072; 0.173]
Motzer 2015	6	54	0.111	[0.042; 0.226]
Gubens 2019	5	45	0.111	[0.037; 0.241]
McDermott 2016	8	70	0.114	[0.051; 0.213]
Galsky 2020	32	270	0.119	[0.082; 0.163]
Powles 2018	55	459	0.120	[0.092; 0.153]
Vogelzang 2020	5	44	0.114	[0.038; 0.246]
Durm 2020	11	93	0.118	[0.061; 0.202]
Gettinger 2016	6	52	0.115	[0.044; 0.234]
Garassino 2018	8	68	0.118	[0.052; 0.219]
Langer 2016	7	59	0.119	[0.049; 0.229]
Lynch 2012	8	67	0.119	[0.053; 0.222]
Antonia 2018	59	475	0.124	[0.096; 0.157]
Rizvi 2020	47	371	0.127	[0.095; 0.165]
McDermott 2015	4	34	0.118	[0.033; 0.275]
Petrylak 2018	12	95	0.126	[0.067; 0.210]
Galsky 2020	59	453	0.130	[0.101; 0.165]
Reck 2021	20	154	0.130	[0.081; 0.193]
Lee 2021	19	145	0.131	[0.081; 0.197]
McKay 2020	11	83	0.133	[0.068; 0.225]
Hellmann 2017	5	39	0.128	[0.043; 0.274]
Powles 2020	47	344	0.137	[0.102; 0.178]
Chen 2020	7	53	0.132	[0.055; 0.253]
Powles 2021	14	103	0.136	[0.076; 0.218]
Nishio 2019	5	38	0.132	[0.044; 0.281]
Motzer 2019	61	434	0.141	[0.109; 0.177]
Balar 2017	52	370	0.141	[0.107; 0.180]
Hellmann 2019	82	576	0.142	[0.115; 0.174]
Theelen 2019	5	37	0.135	[0.045; 0.288]
Paz-Ares 2018	40	278	0.144	[0.105; 0.191]
Govindan 2017	56	388	0.144	[0.111; 0.183]
Pal 2021	5	36	0.139	[0.047; 0.295]
Yamazaki 2017	6	42	0.143	[0.054; 0.285]
Choueiri 2018	8	55	0.145	[0.065; 0.267]
Rini 2019	65	429	0.152	[0.119; 0.189]
Motzer 2020	63	406	0.155	[0.121; 0.194]
Tawbi 2022	57	359	0.159	[0.123; 0.201]
Motzer 2021	58	352	0.165	[0.128; 0.208]
Sugawara 2021	48	273	0.176	[0.133; 0.226]
Eggermont 2018	90	509	0.177	[0.145; 0.213]
Tykodi 2022	9	52	0.173	[0.082; 0.303]
Vuky	66	370	0.178	[0.141; 0.221]
Perets 2021	7	40	0.175	[0.073; 0.328]
Choueiri 2021	91	488	0.186	[0.153; 0.224]
Bellmunt 2021	73	390	0.187	[0.150; 0.229]
McGregor 2020	11	60	0.183	[0.095; 0.304]
Choueiri 2021	60	320	0.188	[0.146; 0.235]
Rini 2019	85	451	0.188	[0.153; 0.228]
Lee 2018	19	100	0.190	[0.118; 0.281]
Bellmunt 2017	52	266	0.195	[0.150; 0.248]
Vaishampayan 2019	12	62	0.194	[0.104; 0.314]
McDermott 2021	33	165	0.200	[0.142; 0.269]
Robert 2019	112	555	0.202	[0.169; 0.238]
Galsky 2018	7	36	0.194	[0.082; 0.360]
Hamid 2019	9	45	0.200	[0.096; 0.346]
Hersh 2011	7	35	0.200	[0.084; 0.369]
Theelen 2019	7	35	0.200	[0.084; 0.369]
Antonia 2016	21	102	0.206	[0.132; 0.297]
Paz-Ares 2021	75	358	0.209	[0.169; 0.255]
Schwarze 2022	7	34	0.206	[0.087; 0.379]
Wolchok 2010	15	71	0.211	[0.123; 0.324]
Powles 2021	66	302	0.219	[0.173; 0.269]
Hamid 2017	39	178	0.219	[0.161; 0.287]
Larkin 2018	59	268	0.220	[0.172; 0.275]
Leighl 2022	33	149	0.221	[0.158; 0.297]
Ascierto 2017	83	364	0.228	[0.186; 0.275]
Powles 2020	78	340	0.229	[0.186; 0.278]
Bajorin 2021	81	351	0.231	[0.188; 0.278]
Leighl 2022	34	148	0.230	[0.165; 0.306]
Powles 2021	81	349	0.232	[0.189; 0.280]
Weber 2017	105	452	0.232	[0.194; 0.274]
Tawbi 2022	83	355	0.234	[0.191; 0.281]
Ascierto 2017	85	362	0.235	[0.192; 0.282]
Hersh 2010	9	39	0.231	[0.111; 0.393]
Dudek 2020	14	60	0.233	[0.134; 0.360]
Planchard 2020	14	60	0.233	[0.134; 0.360]
Grossman 2022	152	639	0.238	[0.205; 0.273]
Lebbé 2019	43	180	0.239	[0.179; 0.308]
Wolchok 2022	75	313	0.240	[0.193; 0.291]
Pal 2021	8	34	0.235	[0.107; 0.412]
Lynch 2012	17	71	0.239	[0.146; 0.355]
Hellmann 2017	9	38	0.237	[0.114; 0.402]
Hodi 2010	32	131	0.244	[0.173; 0.327]
Robert 2020	51	206	0.248	[0.190; 0.312]
Amin 2018	8	33	0.242	[0.111; 0.423]
Hamid 2017	45	179	0.251	[0.190; 0.322]
Zimmer 2020	14	55	0.255	[0.147; 0.390]
Robert 2019	67	256	0.262	[0.209; 0.320]
Lebbé 2019	47	178	0.264	[0.201; 0.335]
Ribas 2016	174	655	0.266	[0.232; 0.301]
Ribas 2012	12	44	0.273	[0.150; 0.428]
Perets 2021	11	40	0.275	[0.146; 0.439]
Postow 2015	13	46	0.283	[0.160; 0.435]
Sharma 2019	30	104	0.288	[0.204; 0.386]
Robert 2011	73	247	0.296	[0.239; 0.357]
McDermott 2021	33	110	0.300	[0.216; 0.395]
Perets 2021	12	40	0.300	[0.166; 0.465]
Jamal 2017	9	30	0.300	[0.147; 0.494]
Ribas 2013	100	325	0.308	[0.258; 0.361]
Albiges 2020	169	547	0.309	[0.270; 0.350]
Sharma 2019	29	92	0.315	[0.222; 0.420]
Long 2021	16	51	0.314	[0.191; 0.459]
Margolin 2012	16	51	0.314	[0.191; 0.459]
Yamazaki 2017	11	35	0.314	[0.169; 0.493]
Weber 2013	13	41	0.317	[0.181; 0.481]
Hammers 2017	15	47	0.319	[0.191; 0.471]
Wolchok 2010	23	71	0.324	[0.218; 0.445]
Hodi 2016	15	46	0.326	[0.195; 0.480]
Sharma 2019	26	78	0.333	[0.231; 0.449]
Weber 2017	152	453	0.336	[0.292; 0.381]
Namikawa 2020	10	30	0.333	[0.173; 0.528]
Grossman 2022	143	420	0.340	[0.295; 0.388]
Postow 2015	33	94	0.351	[0.255; 0.456]
Weber 2016	24	68	0.353	[0.241; 0.478]
Chesney	34	95	0.358	[0.262; 0.463]
Wolchok 2022	113	313	0.361	[0.308; 0.417]
Hammers 2017	17	47	0.362	[0.227; 0.515]
Wolchok 2022	116	311	0.373	[0.319; 0.429]
Long 2018	13	35	0.371	[0.215; 0.551]
Tawbi 2021	39	101	0.386	[0.291; 0.488]
Olson 2021	28	70	0.400	[0.285; 0.524]
Pelster 2021	14	35	0.400	[0.239; 0.579]
Hodi 2016	38	94	0.404	[0.304; 0.510]
Carlino 2020	63	153	0.412	[0.333; 0.494]
Weber 2016	30	70	0.429	[0.311; 0.553]
Eggermont 2016	204	471	0.433	[0.388; 0.479]
Long 2022	27	51	0.529	[0.385; 0.671]
Callahan 2018	50	94	0.532	[0.426; 0.636]
Weber 2016	35	61	0.574	[0.441; 0.700]
Postow 2021	35	60	0.583	[0.449; 0.709]
Patel 2017	56	64	0.875	[0.768; 0.944]
**Total (95% CI)**		**38736**	**0.180**	**[0.164; 0.197]**

Heterogeneity: $Tau^2 = 0.0185$; $Chi^2 = 3021.33$, df = 177 ($P = 0$); $I^2 = 94\%$

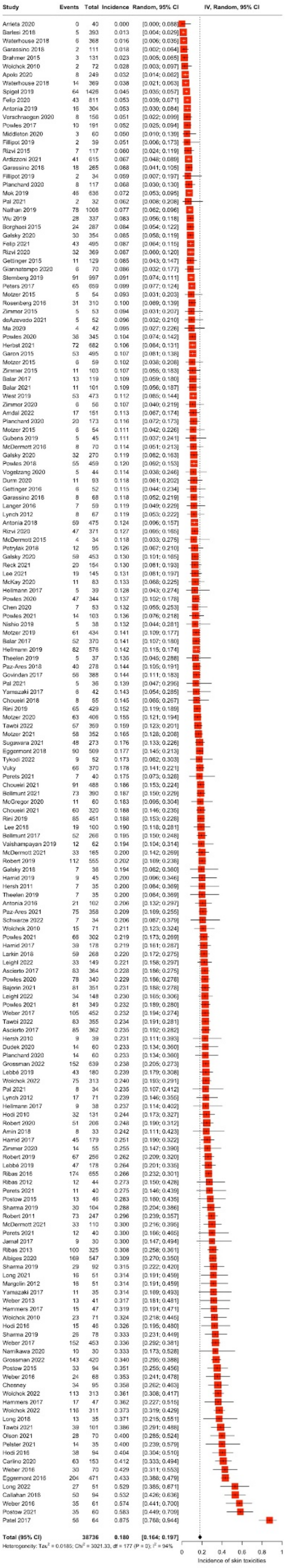

Figure S1. Overall incidence of pruritis across studies.

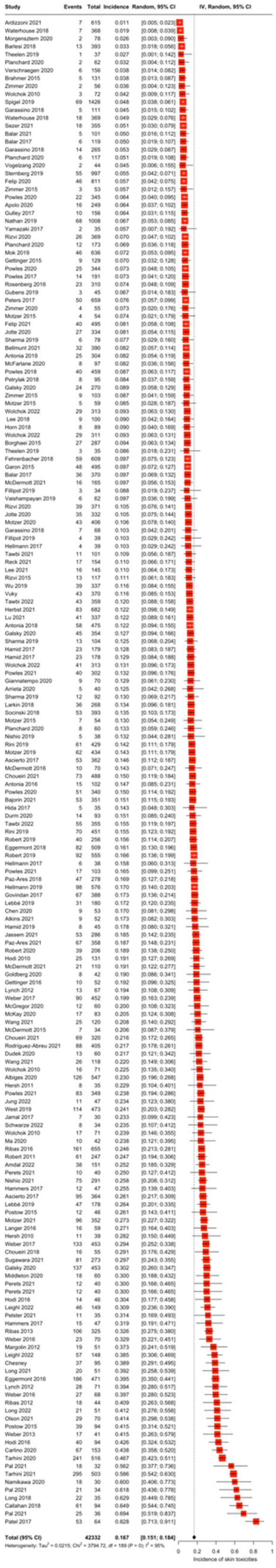

Figure S2. Overall incidence of rash across studies.

Study	Events	Total	Incidence	Fixed, 95% CI
Antonia 2019	1	304	0.003	[0.000; 0.018]
Powles 2020	2	344	0.006	[0.001; 0.021]
Robert 2019	4	256	0.016	[0.004; 0.040]
Zimmer 2020	1	56	0.018	[0.000; 0.096]
Hodi 2010	3	131	0.023	[0.005; 0.065]
Keilholz 2019	1	51	0.020	[0.000; 0.104]
Motzer 2020	12	406	0.030	[0.015; 0.051]
Weber 2013	1	41	0.024	[0.001; 0.129]
Arrieta 2020	1	40	0.025	[0.001; 0.132]
Schwarze 2022	1	34	0.029	[0.001; 0.153]
Zimmer 2020	2	55	0.036	[0.004; 0.125]
Jamal 2017	1	30	0.033	[0.001; 0.172]
Eggermont 2018	24	509	0.047	[0.030; 0.069]
Eggermont 2020	25	509	0.049	[0.032; 0.072]
Wolchok 2022	16	311	0.051	[0.030; 0.082]
Eggermont 2021	9	155	0.058	[0.027; 0.107]
Hersh 2010	2	39	0.051	[0.006; 0.173]
Lebbé 2019	11	178	0.062	[0.031; 0.108]
Hersh 2011	2	35	0.057	[0.007; 0.192]
Hamid 2017	13	178	0.073	[0.039; 0.122]
Namikawa 2020	2	30	0.067	[0.008; 0.221]
Hamid 2017	14	179	0.078	[0.043; 0.128]
Yamazaki 2017	3	42	0.071	[0.015; 0.195]
Wolchok 2022	28	313	0.089	[0.060; 0.127]
Postow 2015	4	46	0.087	[0.024; 0.208]
Tawbi 2022	35	359	0.097	[0.069; 0.133]
deAzevedo 2021	5	52	0.096	[0.032; 0.210]
Tawbi 2022	37	355	0.104	[0.074; 0.141]
Ribas 2016	69	655	0.105	[0.083; 0.131]
Wolchok 2022	33	313	0.105	[0.074; 0.145]
Lebbé 2019	19	180	0.106	[0.065; 0.160]
Larkin 2018	29	268	0.108	[0.074; 0.152]
Postow 2015	10	94	0.106	[0.052; 0.187]
Long 2018	4	35	0.114	[0.032; 0.267]
Long 2021	6	51	0.118	[0.044; 0.239]
Robert 2019	71	555	0.128	[0.101; 0.159]
Long 2022	7	51	0.137	[0.057; 0.263]
Callahan 2018	15	94	0.160	[0.092; 0.250]
Robert 2020	34	206	0.165	[0.117; 0.223]
Carlino 2020	31	153	0.203	[0.142; 0.275]
**Total (95% CI)**		**7693**	**0.066**	**[0.060; 0.072]**

Heterogeneity: $Tau^2 = 0.0078$; $Chi^2 = 270.04$, df = 39 ($P < 0.01$); $I^2 = 86\%$

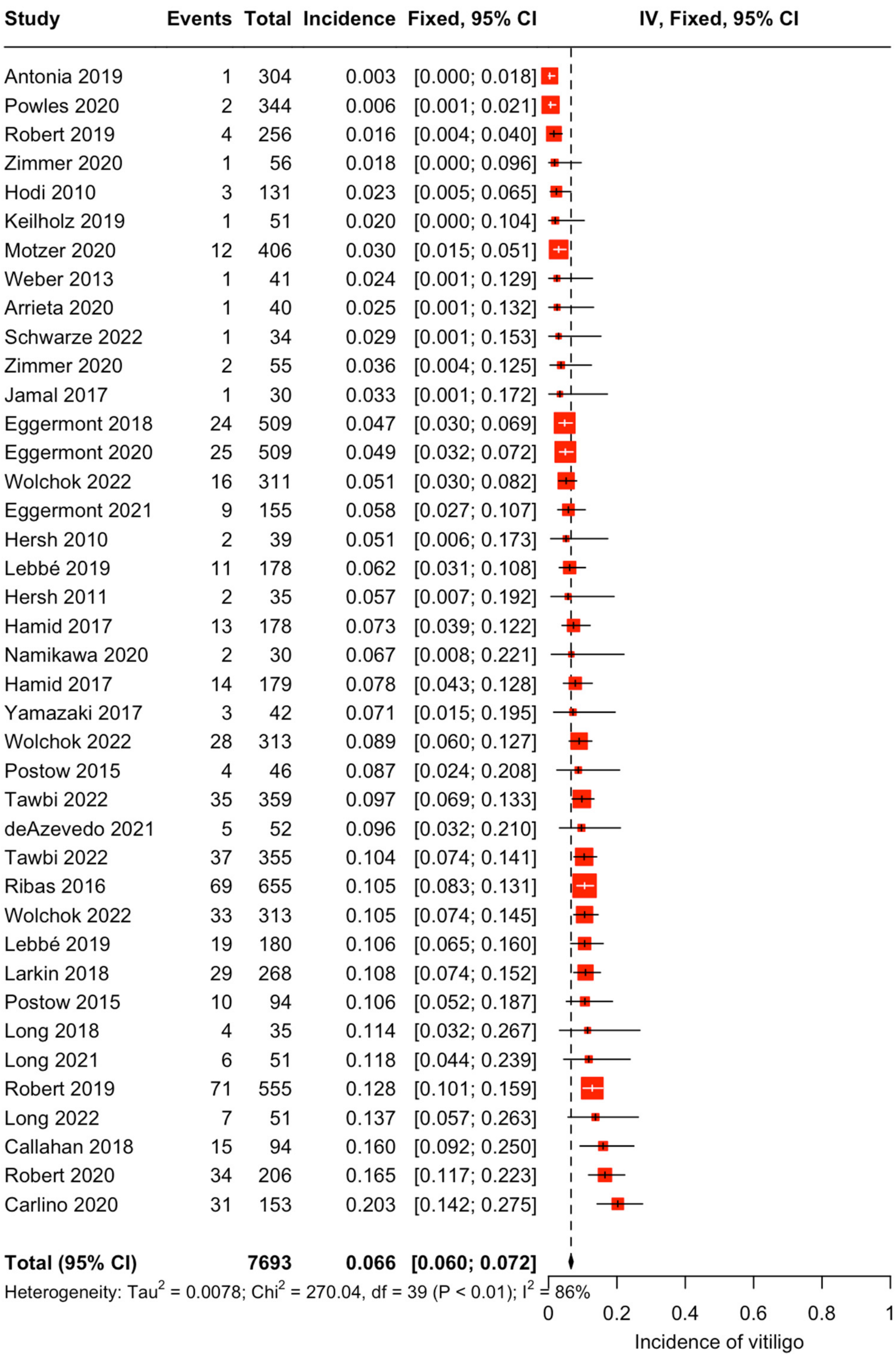

Figure S3. Overall incidence of vitiligo across studies.

Study	Events	Total	Incidence	Random, 95% CI
Eggermont 2020	30	509	0.059	[0.040; 0.083]
Waterhouse 2018	26	368	0.071	[0.047; 0.102]
Waterhouse 2020	10	125	0.080	[0.039; 0.142]
Waterhouse 2018	42	369	0.114	[0.083; 0.151]
Waterhouse 2020	16	127	0.126	[0.074; 0.197]
Wolchok 2010	9	72	0.125	[0.059; 0.224]
Spigel 2019	201	1426	0.141	[0.123; 0.160]
Wolchok 2022	49	313	0.157	[0.118; 0.202]
Gettinger 2015	20	129	0.155	[0.097; 0.229]
Hayashi 2022	8	52	0.154	[0.069; 0.281]
Wolchok 2022	50	311	0.161	[0.122; 0.206]
Fehrenbacher 2018	103	609	0.169	[0.140; 0.201]
Felip 2020	143	811	0.176	[0.151; 0.204]
Wu 2019	71	337	0.211	[0.168; 0.258]
Zimmer 2015	11	53	0.208	[0.108; 0.341]
Lu 2021	73	337	0.217	[0.174; 0.264]
Motzer 2015	13	59	0.220	[0.123; 0.347]
Motzer 2015	12	54	0.222	[0.120; 0.356]
Galsky 2020	64	270	0.237	[0.188; 0.292]
Wolchok 2022	76	313	0.243	[0.196; 0.294]
Gettinger 2016	13	52	0.250	[0.140; 0.389]
Nathan 2019	266	1008	0.264	[0.237; 0.292]
Eggermont 2016	127	471	0.270	[0.230; 0.312]
Zimmer 2020	15	56	0.268	[0.158; 0.403]
Motzer 2020	113	406	0.278	[0.235; 0.325]
Motzer 2015	15	54	0.278	[0.165; 0.416]
Eggermont 2018	144	509	0.283	[0.244; 0.324]
Hida 2017	10	35	0.286	[0.146; 0.463]
Hellmann 2019	196	576	0.340	[0.302; 0.381]
Ascierto 2017	129	362	0.356	[0.307; 0.408]
Govindan 2017	139	388	0.358	[0.310; 0.408]
Hellmann 2017	14	39	0.359	[0.212; 0.528]
Larkin 2018	104	268	0.388	[0.329; 0.449]
Hellmann 2017	15	38	0.395	[0.240; 0.566]
Hamid 2012	16	40	0.400	[0.249; 0.567]
Paz-Ares 2021	145	358	0.405	[0.354; 0.458]
Bajorin 2021	143	351	0.407	[0.356; 0.461]
Powles 2021	127	302	0.421	[0.364; 0.478]
Sharma 2019	44	104	0.423	[0.327; 0.524]
Ascierto 2017	156	364	0.429	[0.377; 0.481]
Hersh 2011	15	35	0.429	[0.263; 0.606]
Hodi 2010	57	131	0.435	[0.349; 0.524]
Sharma 2019	34	78	0.436	[0.324; 0.553]
Lebbé 2019	79	180	0.439	[0.365; 0.515]
Weber 2017	201	452	0.445	[0.398; 0.492]
Wolchok 2010	32	71	0.451	[0.332; 0.573]
Sharma 2019	42	92	0.457	[0.352; 0.564]
Wolchok 2010	33	71	0.465	[0.345; 0.587]
DiGiacomo	40	86	0.465	[0.357; 0.576]
Hersh 2010	19	39	0.487	[0.324; 0.652]
Hammers 2017	23	47	0.489	[0.341; 0.639]
O'Day 2010	76	155	0.490	[0.409; 0.572]
Hamid 2011	21	42	0.500	[0.342; 0.658]
Albiges 2020	279	547	0.510	[0.467; 0.553]
Lebbé 2019	94	178	0.528	[0.452; 0.603]
Zimmer 2020	32	55	0.582	[0.441; 0.713]
Powles 2021	203	349	0.582	[0.528; 0.634]
Postow 2015	27	46	0.587	[0.432; 0.730]
Hammers 2017	28	47	0.596	[0.443; 0.736]
Weber 2017	271	453	0.598	[0.551; 0.644]
Piulats 2021	32	52	0.615	[0.470; 0.747]
Weber 2009	39	57	0.684	[0.548; 0.801]
Postow 2015	67	94	0.713	[0.610; 0.801]
Long 2018	26	35	0.743	[0.567; 0.875]
Amin 2018	26	33	0.788	[0.611; 0.910]
**Total (95% CI)**		**15850**	**0.348**	**[0.306; 0.391]**

Heterogeneity: $Tau^2 = 0.0305$; $Chi^2 = 1937.46$, df = 64 (P = 0); $I^2 = 97\%$

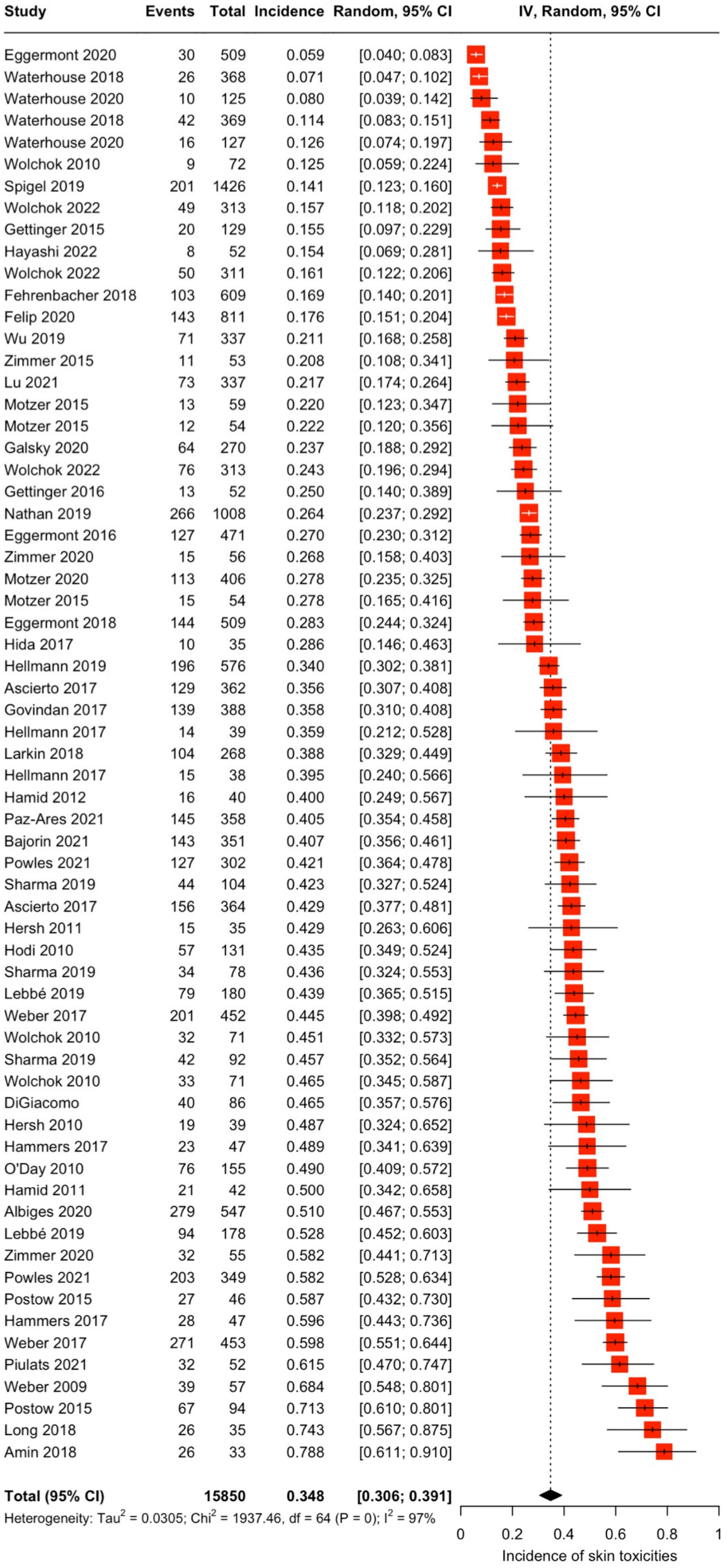

Figure S4. Overall incidence of cirAEs across studies.

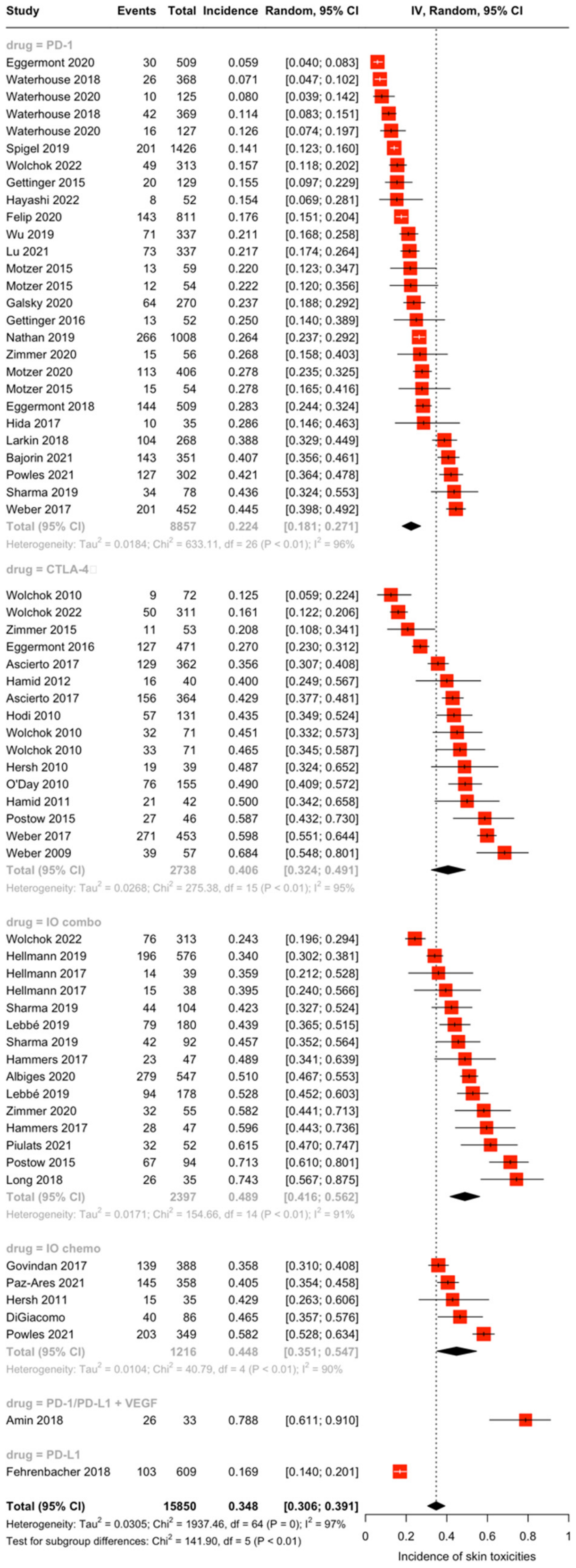

Figure S5. Overall incidence of cirAEs by drug class groupings.

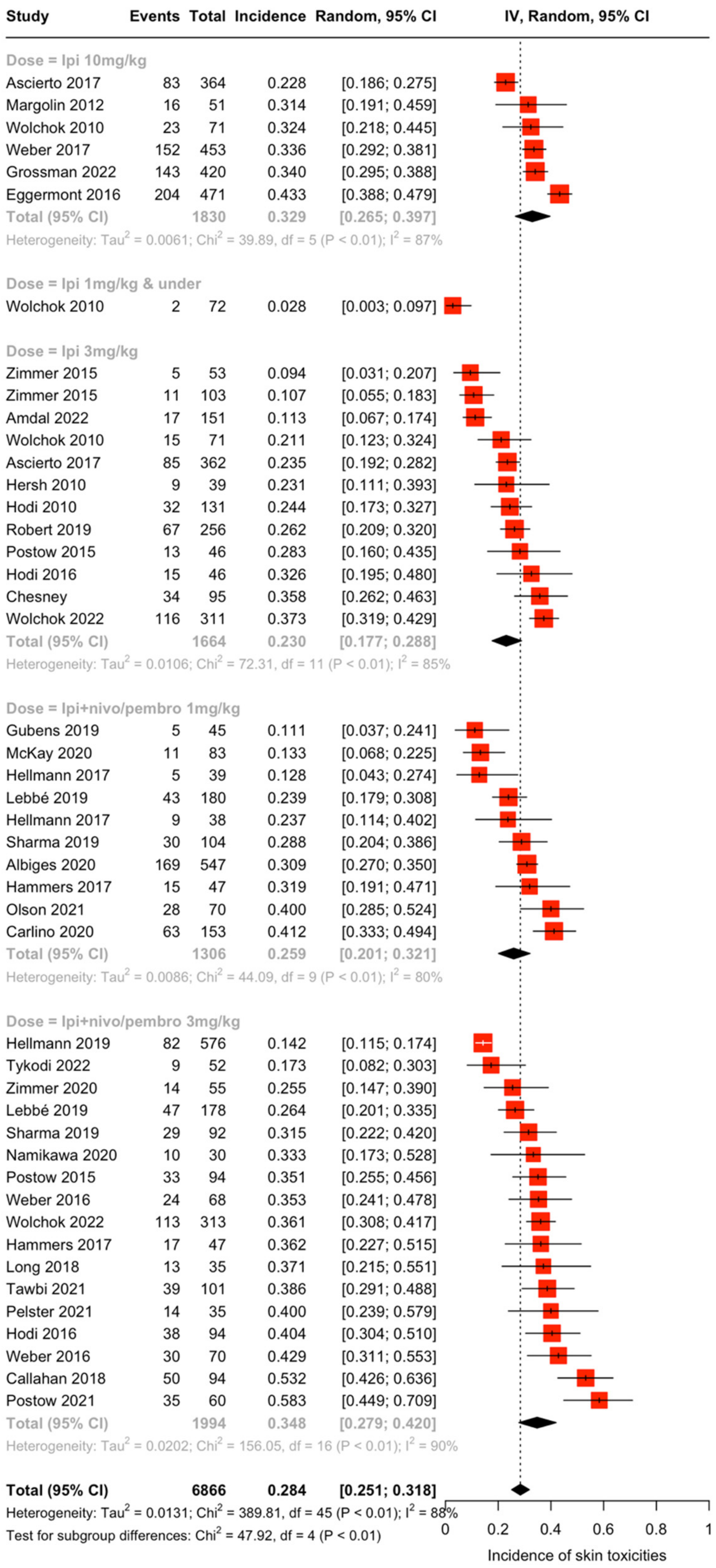

Figure S6. Overall incidence of pruritis by ipilimumab dose groupings.

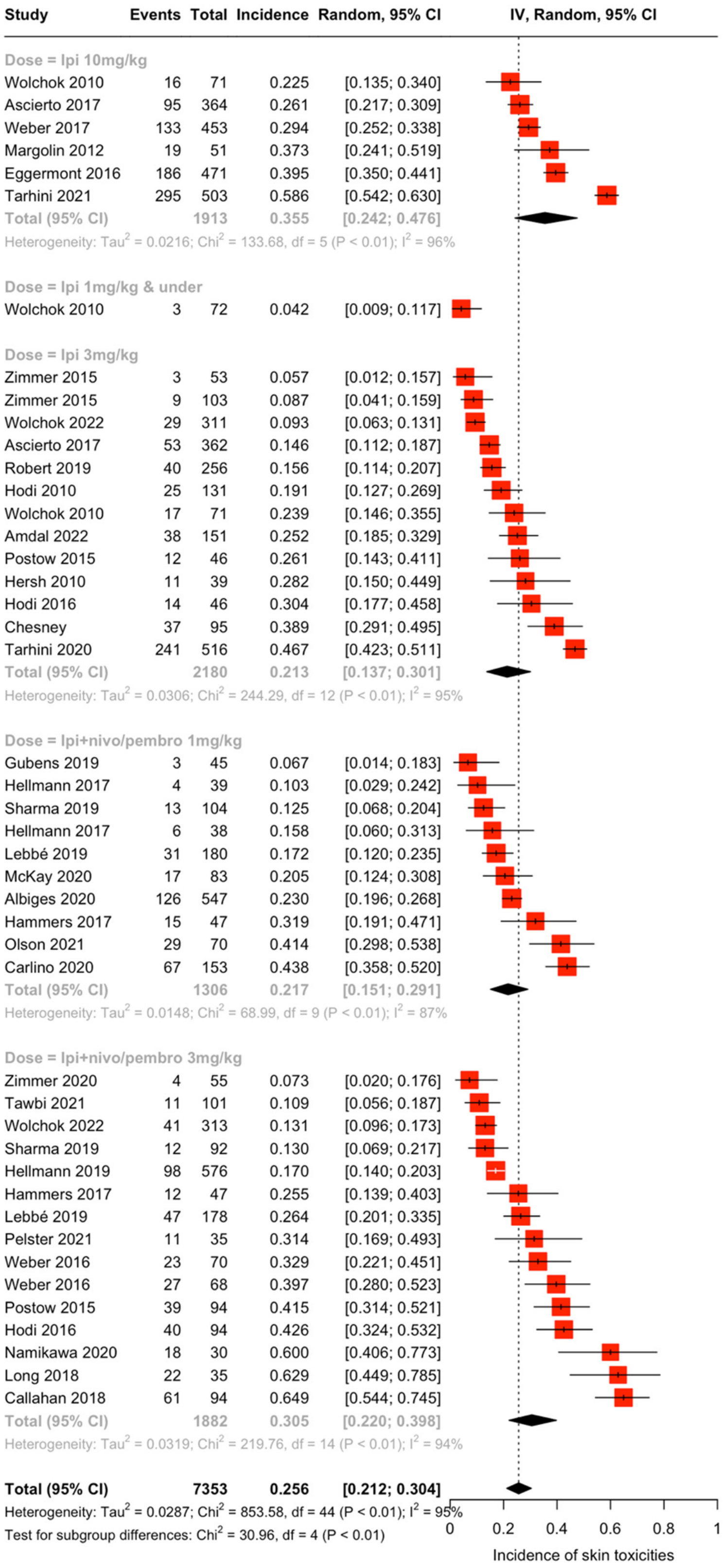

Figure S7. Overall incidence of rash by ipilimumab dose groupings.

Study	Events	Total	Incidence	Fixed, 95% CI
Dose = Ipi 3mg/kg				
Robert 2019	4	256	0.016	[0.004; 0.040]
Hodi 2010	3	131	0.023	[0.005; 0.065]
Wolchok 2022	16	311	0.051	[0.030; 0.082]
Hersh 2010	2	39	0.051	[0.006; 0.173]
Postow 2015	4	46	0.087	[0.024; 0.208]
Total (95% CI)		**783**	**0.032**	**[0.019; 0.046]**
Heterogeneity: $Tau^2 = 0.0024$; $Chi^2 = 9.39$, df = 4 (P = 0.05); $I^2 = 57\%$				
Dose = Ipi+nivo/pembro 1mg/kg				
Lebbé 2019	19	180	0.106	[0.065; 0.160]
Carlino 2020	31	153	0.203	[0.142; 0.275]
Total (95% CI)		**333**	**0.147**	**[0.111; 0.187]**
Heterogeneity: $Tau^2 = 0.0076$; $Chi^2 = 6.04$, df = 1 (P = 0.01); $I^2 = 83\%$				
Dose = Ipi+nivo/pembro 3mg/kg				
Zimmer 2020	2	55	0.036	[0.004; 0.125]
Lebbé 2019	11	178	0.062	[0.031; 0.108]
Namikawa 2020	2	30	0.067	[0.008; 0.221]
Wolchok 2022	28	313	0.089	[0.060; 0.127]
Postow 2015	10	94	0.106	[0.052; 0.187]
Long 2018	4	35	0.114	[0.032; 0.267]
Callahan 2018	15	94	0.160	[0.092; 0.250]
Total (95% CI)		**799**	**0.085**	**[0.066; 0.106]**
Heterogeneity: $Tau^2 = 0.0013$; $Chi^2 = 9.17$, df = 6 (P = 0.16); $I^2 = 35\%$				
**Total (95% CI)**		**1915**	**0.069**	**[0.057; 0.081]**
Heterogeneity: $Tau^2 = 0.0080$; $Chi^2 = 68.24$, df = 13 (P < 0.01); $I^2 = 81\%$				
Test for subgroup differences: $Chi^2 = 43.64$, df = 2 (P < 0.01)				

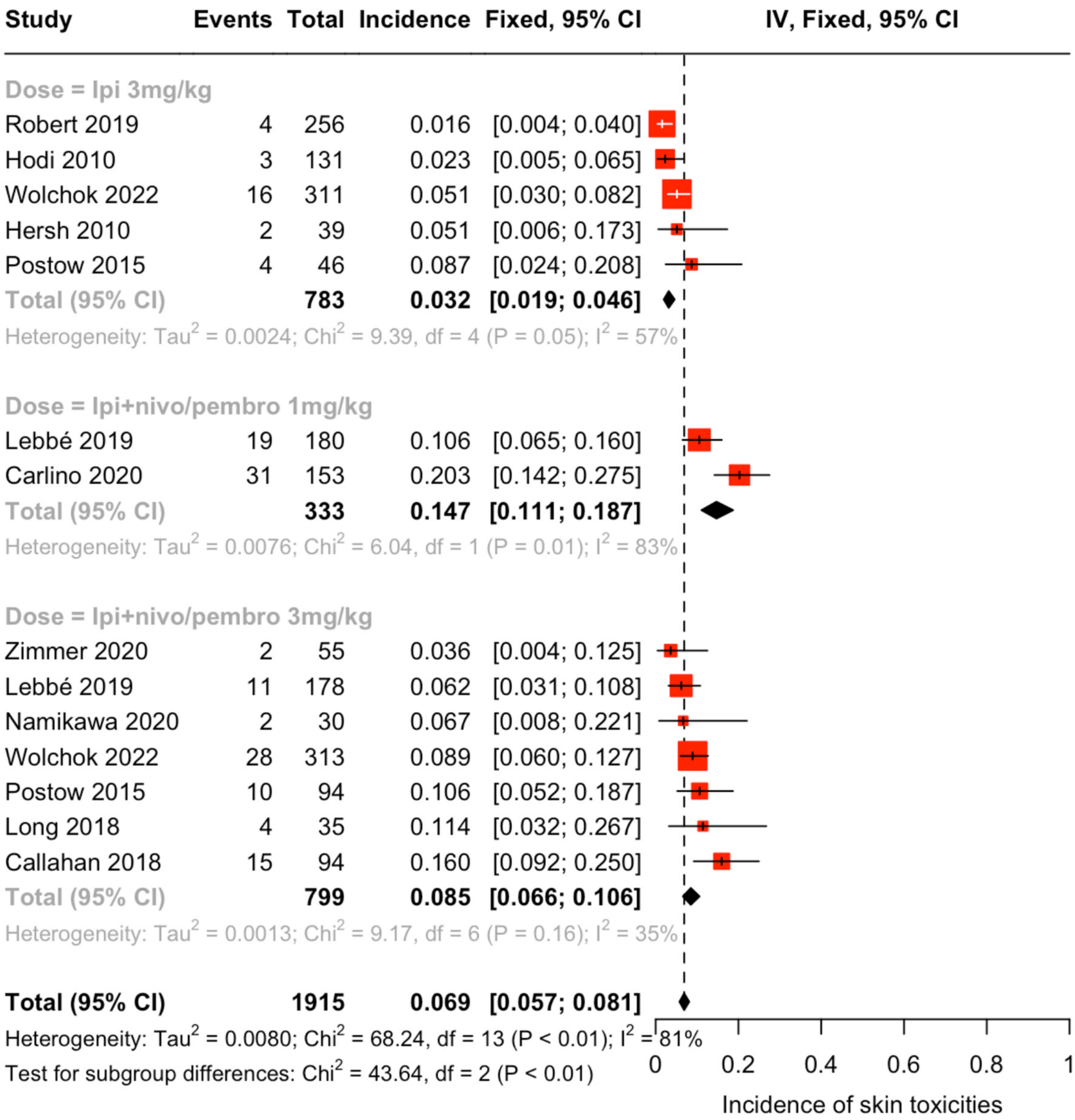

Figure S8. Overall incidence of vitiligo by ipilimumab dose groupings.

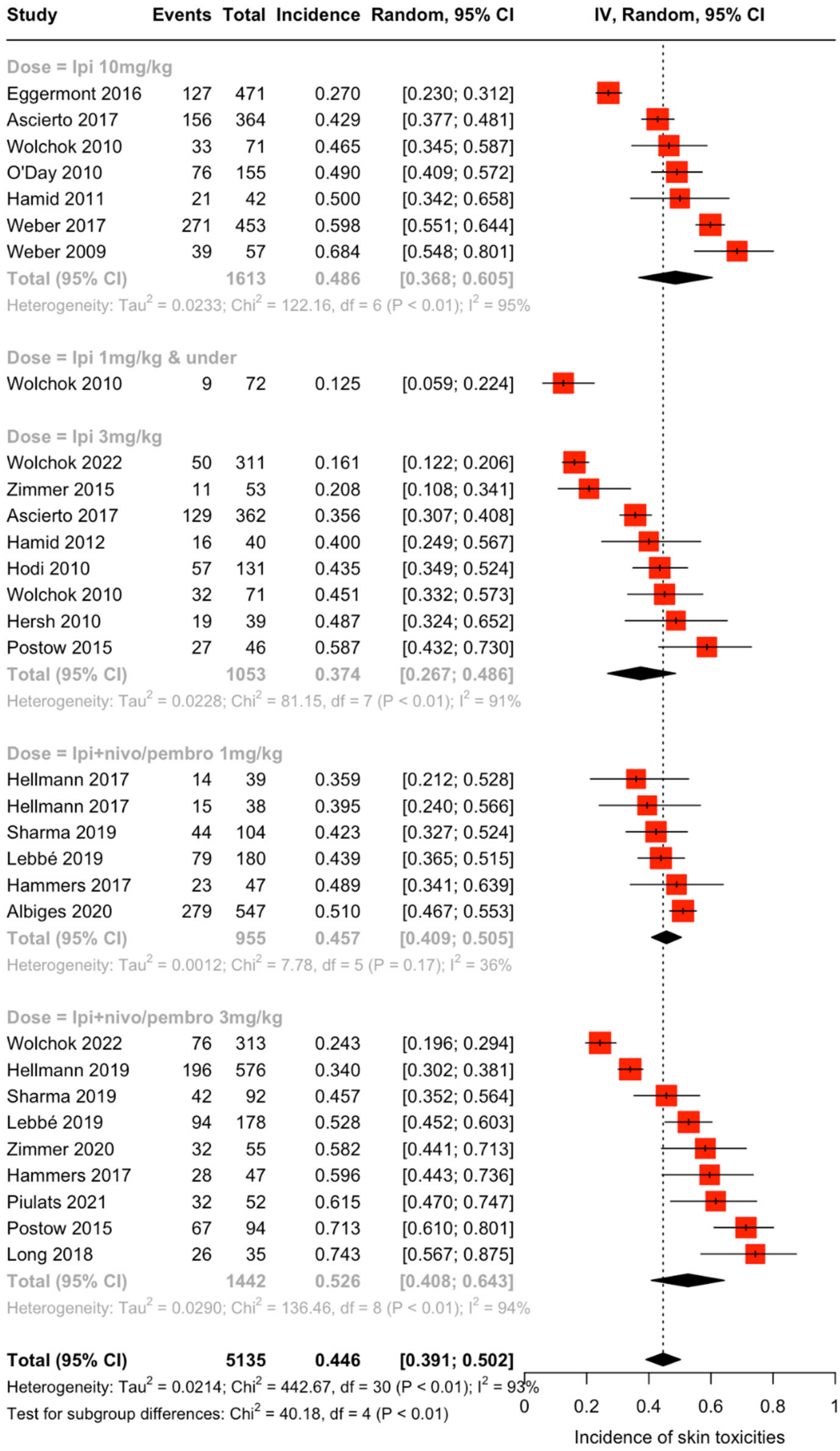

Figure S9. Overall incidence of cirAEs by ipilimumab dose groupings.

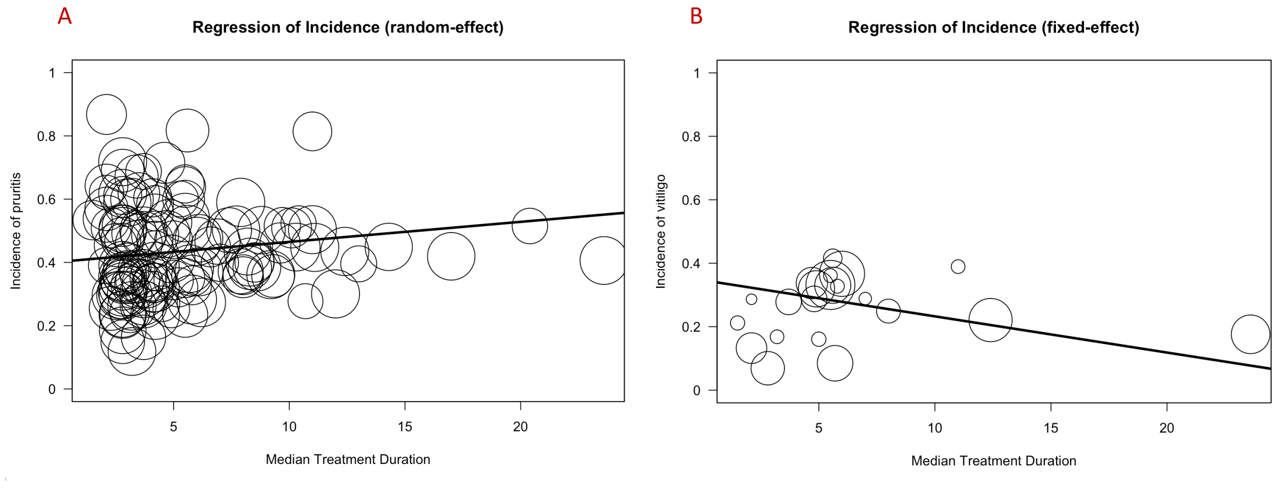

Figure S10: Bubble plots demonstrating the estimated regression slope for. incidence of pruritis (A) and vitiligo (B) and duration of treatment (median months).

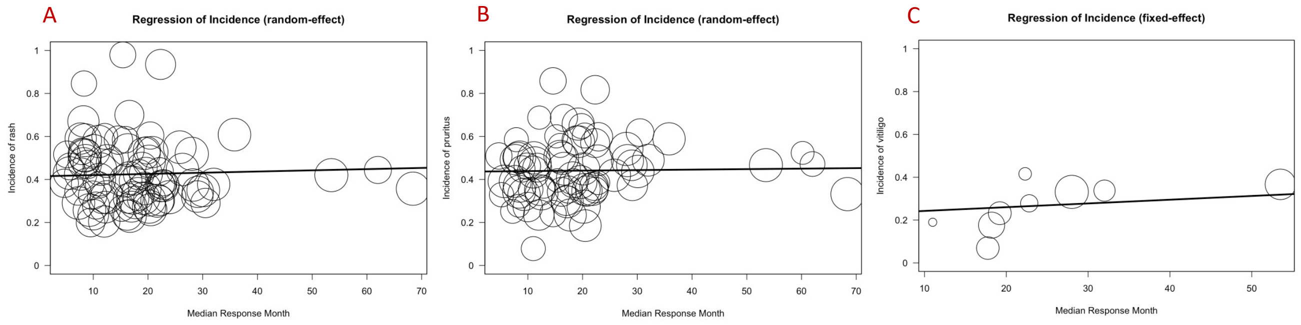

Figure S11. Bubble plot demonstrating the estimated regression slope for incidence of rash (A), pruritis (B), and vitiligo (C) and duration of response. Duration of response was positively correlated with incidence of rash, pruritis, and vitiligo though these associations were not significant.

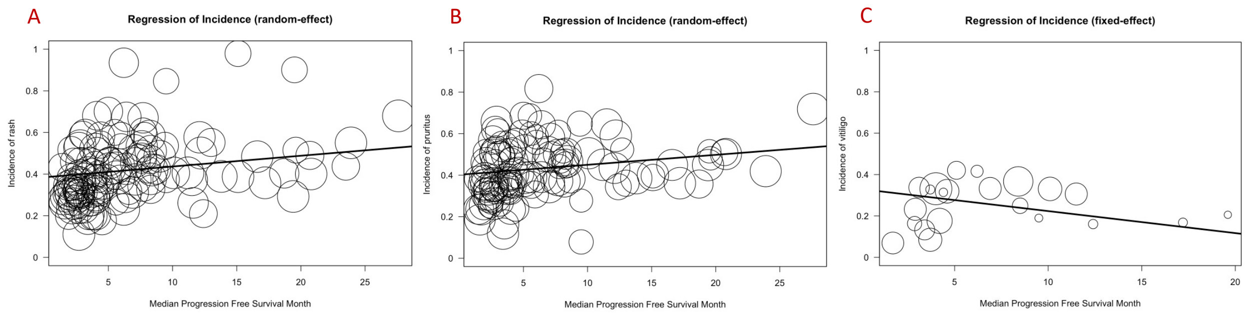

Figure S12. Bubble plot demonstrating the estimated regression slope for incidence of rash (A), pruritis (B), and vitiligo (C) and progression-free survival. Progression-free survival was positively associated with incidence of pruritis and rash, and negatively associated with vitiligo. These associations were significant. An expected rise in incidence of pruritis and rash per unit increase in progression-free survival was 0.005% (p=0.0207) and 0.005% (p=0.0351), respectively. An expected decrease in incidence of vitiligo per additional month of progression-free survival was 0.011% (p=0.0029). .
